# Correlation of the Dzyaloshinskii–Moriya interaction with Heisenberg exchange and orbital asphericity

**DOI:** 10.1038/s41467-018-04017-x

**Published:** 2018-04-25

**Authors:** Sanghoon Kim, Kohei Ueda, Gyungchoon Go, Peong-Hwa Jang, Kyung-Jin Lee, Abderrezak Belabbes, Aurelien Manchon, Motohiro Suzuki, Yoshinori Kotani, Tetsuya Nakamura, Kohji Nakamura, Tomohiro Koyama, Daichi Chiba, Kihiro. T. Yamada, Duck-Ho Kim, Takahiro Moriyama, Kab-Jin Kim, Teruo Ono

**Affiliations:** 1 Institute for Chemical Research, Kyoto University, Uji, Kyoto 611-0011 Japan; 2 Department of Physics, University of Ulsan, Ulsan, 44610 Korea; 3 Department of Materials Science and Engineering, Massachusetts Institute of Technology, Cambridge, MA 02139 USA; 4 Department of Materials Science & Engineering, Korea University, Seoul, 02841 Korea; 5 KU-KIST Graduate School of Converging Science and Technology, Korea University, Seoul, 02841 Korea; 6 Physical Science and Engineering Division (PSE), King Abdullah University of Science and Technology (KAUST), Thuwal, 23955-6900 Saudi Arabia; 7 Japan Synchrotron Radiation Research Institute (JASRI), Sayo, Hyogo 679-5198 Japan; 8 Department of Physics Engineering, Mie University, Tsu, Mie 514-8507 Japan; 9 Department of Applied Physics, The University of Tokyo, Bunkyo, Tokyo 113-8656 Japan; 10 Department of Physics, Korea Advanced Institute of Science and Technology, Daejeon, 34141 Korea; 11 Center for Spintronics Research Network (CSRN), Graduate School of Engineering Science, Osaka University, Osaka, 560-8531 Japan

## Abstract

Chiral spin textures of a ferromagnetic layer in contact to a heavy non-magnetic metal, such as Néel-type domain walls and skyrmions, have been studied intensively because of their potential for future nanomagnetic devices. The Dyzaloshinskii–Moriya interaction (DMI) is an essential phenomenon for the formation of such chiral spin textures. In spite of recent theoretical progress aiming at understanding the microscopic origin of the DMI, an experimental investigation unravelling the physics at stake is still required. Here we experimentally demonstrate the close correlation of the DMI with the anisotropy of the orbital magnetic moment and with the magnetic dipole moment of the ferromagnetic metal in addition to Heisenberg exchange. The density functional theory and the tight-binding model calculations reveal that inversion symmetry breaking with spin–orbit coupling gives rise to the orbital-related correlation. Our study provides the experimental connection between the orbital physics and the spin–orbit-related phenomena, such as DMI.

## Abstract (web summary)

Dzyaloshinskii–Moriya interaction (DMI) is one of the key factors to control the chiral spin textures in spintronic applications. Here the authors demonstrate the correlation of the DMI with the anisotropy of the orbital magnetic moment and magnetic dipole moment in Pt/Co/MgO ultrathin trilayers.

## Introduction

Chiral interaction between two atomic spins owing to a strong spin–orbit coupling (SOC), which is known as the Dzyaloshinskii–Moriya interaction (DMI), has attracted intense interest^1,2^. In particular, it has been demonstrated that the DMI at the interface between ferromagnetic (FM) and non-magnetic heavy metals (HMs) plays a major role for the formation of chiral spin textures, such as skyrmions^3,4^ and homochiral Néel-type domain walls (DWs)^5–7^, which are attractive for the development of future information storage technology^8^. Understanding the microscopic origin of the DMI is indispensable for the realization of such chiral spin textures^9,10^. It has been reported that the proximity-induced magnetic moment in HM layers is critical to promote the DMI^11^. However, this proximity effect is still controversial because it has been also reported that the induced magnetic moment has no direct correlation with the DMI in the case of the Co/Pt system^12,13^. The scattering of spin-polarized electrons on spin–orbit coupled impurities is known to give rise to the DMI in spin-glass systems as a microscopic viewpoint^14,15^. However, the orbital hybridization between the spin–orbit coupled ions and the magnetic matrix was explicitly neglected in the theory, which becomes problematic when considering transition metal interfaces. On the other hand, theories have predicted that SOC combined with inversion symmetry breaking (ISB) naturally introduces a chirality to conduction electron spins in equilibrium and the interfacial DMI at an FM/HM interface is related to this spin chirality^16–20^. It has also been reported that the spin chirality is a manifestation of the chirality of the orbital magnetism in strongly spin–orbit coupled systems with ISB^21,22^. These previous studies suggest a possible microscopic origin of the interfacial DMI, which has remained experimentally unaddressed so far.

Here we discuss the microscopic origin of the interfacial DMI with experimental and theoretical studies as follows: First, we show the temperature dependence of the DMI for a Pt/Co/MgO trilayer, which is one of the standard structures used for the studies of the DMI^7,12,23^, using the extended droplet model^24^. We find that the DMI increases with decreasing temperature in a range from 300 to 100 K. In general, the electron–phonon interaction promotes thermally induced hopping between nearest neighbours when increasing the temperature^25^. As a result, it is expected that the difference between in-plane and out-of-plane hopping energies is reduced upon temperature increase. Therefore, changing the temperature of the system allows for charge redistribution between in-plane and out-of-plane orbitals while preserving the integrity of electronic states of the trilayer unlike other interface control methods such as ion irradiation^26^ or thermal annealing technique^27^, which may cause a permanent atomic rearrangement and thus induce undesired extrinsic effects. To discuss this temperature dependence of the DMI, that of the spin (***m***_s_) and orbital (***m***_o_) magnetic moments of Co and Pt is studied by X-ray magnetic circular dichroism (XMCD) spectroscopy. We find that ***m***_s_ values of Co and Pt show temperature dependences due to change in Heisenberg exchange. Furthermore, the intra-atomic magnetic dipole moment (***m***_D_), which is due to the asymmetric spin density distribution (*f*_SD_)^28,29^, displays strong temperature dependence, suggesting a sizable modification of the charge distribution between the in-plane and the out-of-plane *d*-orbitals under temperature variation. We also find that the out-of-plane orbital moment (${\boldsymbol{m}}_{\mathrm{o}}^ \bot$) shows large temperature dependence while in-plane orbital moment (${\boldsymbol{m}}_{\mathrm{o}}^\parallel$) does not, revealing a close connection between the anisotropy of ***m***_0_ (orbital anisotropy) and the DMI. The ab initio and the tight-binding model calculations suggest that the ISB-dependent electron hopping, which gives rise to the asymmetric charge distribution at the interface of the FM/HM, is a possible microscopic origin of the correlation between the orbital anisotropy and the DMI.

## Results

### Temperature dependence of the DMI in the Pt/Co/MgO trilayer

**Fig. 1:**
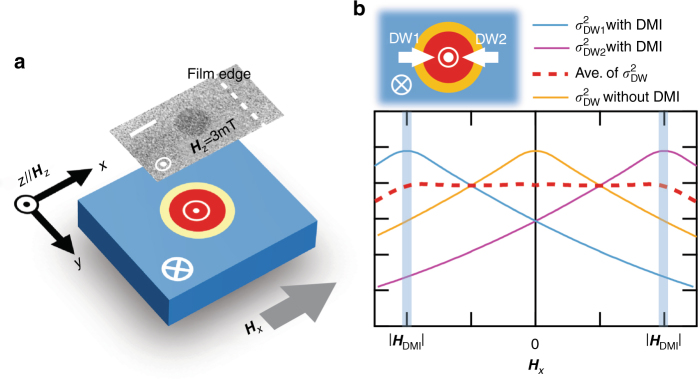
Droplet nucleation with DMI. **a** Schematic image of a magnetic droplet in a ferromagnetic medium under ***H***_*z*_ and ***H***_*x*_. Inset shows a magneto-optical Kerr effect (MOKE) image to prove the droplet nucleation inside the Pt/Co/MgO microstrip. The white bar is a scale bar of 5 μm. **b** Schematic diagrams of ${\mathrm{\sigma }}_{{\mathrm{DW}}}^2$ in terms of ***H***_*x*_. Inset shows the magnetization alignments of the DW1 and DW2 in the DW of the droplet. The thresholds in the curve of averaged ${\mathrm{\sigma }}_{{\mathrm{DW}}}^2$ are highlighted with blue shades

The temperature dependence of the DMI-induced effective field (***H***_DMI_) of the Pt (2 nm)/Co (0.5  nm)/MgO (2 nm) trilayer is determined by measuring the nucleation field (***H***_n_) applied along the in-plane (***H***_*x*_) and out-of-plane (***H***_*z*_) direction as schematically shown in Fig. 1a. The measured ***H***_n_ values are analysed with the extended droplet model^24^. Since ***H***_n_ is proportional to square of DW energy (${\mathrm{\sigma }}_{{\mathrm{DW}}}^2$), the *σ*_DW_ is a crucial parameter for the nucleation^24,30^. In case of the droplet with sizable DMI, DW magnetizations are aligned in the radial direction. When we consider the two DW magnetizations with respect to ***H***_*x*_, DW1 and DW2 (see the inset of Fig. 1b), ${\mathrm{\sigma }}_{{\mathrm{DW}}}^2$ of the DW1 and the DW2, respectively, follows the blue and the red curves in terms of ***H***_*x*_ due to DMI as illustrated in Fig. 1b^31^. Assuming that total DW energy mainly depends on the two magnetizations at the DW of a droplet with respect to ***H***_*x*_, the total ${\mathrm{\sigma }}_{{\mathrm{DW}}}^2$ follows the trend of the purple-dotted line. Note that there is a threshold where the ${\mathrm{\sigma }}_{{\mathrm{DW}}}^2$ starts to become ***H***_*x*_ dependent. Our previous report showed that the extended droplet model allows us to estimate the DMI-induced field from the threshold field^24^. In spite of such one-dimensional approximation, we have demonstrated that the fitting gives reliable value of the DMI energy density (*D*).

**Fig. 2:**
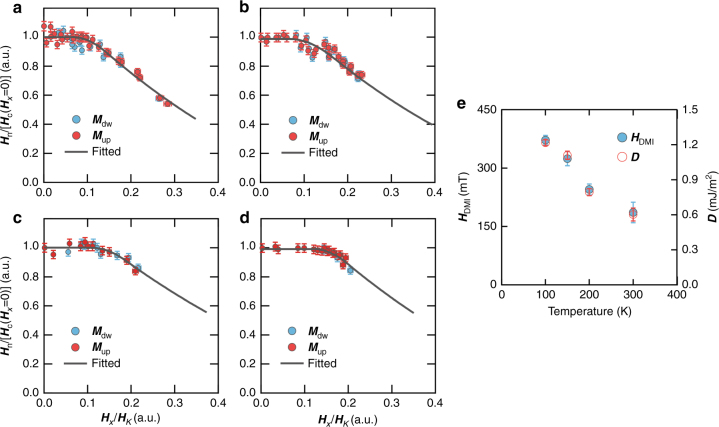
***H***_DMI_ measurement from the *H*_n_ of the droplet. **a**–**d** The ***H***_n_/***H***_sw_(***H***_*x*_=0) vs ***H***_*x*_/***H***_*K*_ plots measured at 300, 200, 150 and 100 K, respectively. The grey solid lines are the best fitting results using the droplet model. Here the vertical axis is normalized by the nucleation field ***H***_s__w_(***H***_*x*_=0) at ***H***_*x*_=0 and the horizontal axis is normalized by the effective perpendicular anisotropy field ***H***_*K*_. **e** Plots of ***H***_DMI_ and ***D*** in terms of *T*. The error bars are based on the standard deviation of the ***H***_DMI_ distribution from the best fitting

The measured ***H***_n_ as a function of ***H***_*x*_ shows a threshold arising from the DMI as predicted by the extended droplet model^24^ (see also Supplementary Note 1, 2 and Method section for details about the analysis and the measurement). Figures 2a–d show ***H***_n_/***H***_SW_(***H***_*x*_=0) as a function of ***H***_*x*_/***H***_*K*_ at various temperatures (a, *T*=300 K; b, 200 K; c, 150 K and d, 100 K). Here ***H***_n_ and ***H***_*x*_ are normalized by the switching field at ***H***_*x*_=0 [***H***_SW_(***H***_*x*_=0)] and by the anisotropy field (***H***_*K*_), respectively. Those normalized values allow us to clearly confirm the DMI-dependent threshold with ruling out the temperature-dependent characteristics of ***H***_*K*_ and the ***H***_SW_(***H***_*x*_=0). The temperature-dependent ***H***_DMI_ can be determined from the best fitting using the extended droplet model; ***H***_DMI_ at 300, 200, 150 and 100 K are 166±50, 245±45, 324±15 and 372±30 mT, respectively (Fig. 2e). The temperature-dependent ***D*** is readily calculated from ***H***_DMI_ and *Δ*via ${\boldsymbol{H}}_{{\mathrm{DMI}}} = {\boldsymbol{D}}/\mu _0M_{\mathrm{s}}\varDelta$, where *μ*_0_ is the permeability, *M*_s_ is the saturation magnetization and *Δ* is the DW width^6^. *Δ* and *M*_s_ values in terms of the temperature are listed in Table 1. We find that ***D*** has a strong temperature dependence as shown in Fig. 2e; ***D*** increases by a factor of 2.2 as the temperature decreases from 300 to 100 K.

**Table 1:** Parameters to estimate the DW energy

Temperature	*M*_s_ (MA/m)	*K*_U_ (MJ/m^3^)	*A* (pJ/m)	*∆* (nm)	*K*_D_ (10 kJ/m^3^)	*σ*_0_ (mJ/m^2^)
300	1.06	0.83±0.01	5.85±0.1	2.7±0.1	9.9±0.4	8.80±0.4
200	1.22	1.26±0.01	7.76±0.1	2.5±0.1	9.82±2.0	12.5±0.3
150	1.28	1.37±0.01	8.81±0.1	2.5±0.1	9.82±2.0	13.9±0.2
100	1.33	1.60±0.20	9.22±0.1	2.4±0.2	9.15±2.0	15.4±0.2

*∆*, the domain wall anisotropy (*K*_D_), and the Bloch-type DW energy (σ_0_) values obtained from the best fitting of ***H***_n_(***H***_*x*_)/[***H***_sw_(***H***_x_=0)] vs ***H***_*x*_/***H***_*K*_ plots using the extended droplet model. *K*_D_ is DW anisotropy energy, representing the magnetostatic energy difference between Bloch DW and Néel DW^41^. The values are comparable with previous reported values in ref. ^28^. *M*_s_ values were measured using a superconducting quantum interference device magnetometer. Details about estimation of *A* are given in Supplementary Note 3.

Heisenberg exchange is one of key parameters to understand the microscopic origin of DMI^2,32^. From the temperature dependence of *M*_s_, the exchange stiffness constant (*A*) values were quantitatively obtained as detailed in Supplementary Note 3. As listed in Table 1, we find a clear correlation of the Heisenberg exchange with DMI; the *A* value increases by 57% when the temperature decreases from 300K to 100 K while DMI shows 100% increase. Based on Moriya’s theory, DMI requires three ingredients: (i) SOC, (ii) magnetic exchange, and (iii) ISB. Therefore, we can consider two parameters in addition to Heisenberg exchange: one is the proximity-induced magnetism of the non-magnetic element, which is related to the source ii, and the other is the hybridization between 3*d* and 5*d* orbitals, which is related to the sources i and iii. In order to find how DMI correlated with the induced moment in the Pt layer and the orbital structure of Co, XMCD studies were performed using the soft and hard X-ray as discussed in the following sections.

### Temperature dependence of the proximity-induced magnetic moment of Pt

**Fig. 3:**
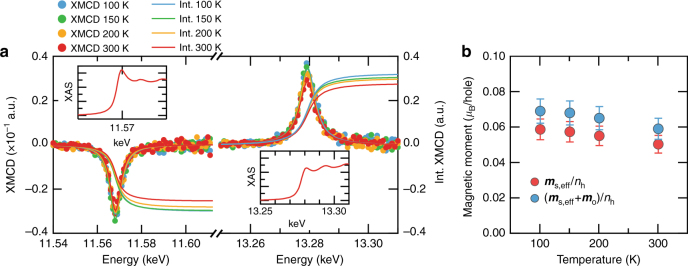
Temperature dependence of the proximity-induced moment in the Pt layer. **a** The XMCD and integrated XMCD spectra at the Pt *L*_3_ and *L*_2_ edges in terms of temperature. The XMCD spectra were fitted by the Lorentzian function, and the fitted curves were integrated to get the integrated XMCD spectra. The insets are the XAS spectra at the Pt *L*_3_ and *L*_2_ edges. **b** Temperature dependence of the ${\boldsymbol{m}}_{\mathrm{s}}^{{\mathrm{eff}}}$ and ${\boldsymbol{m}}_{{\mathrm{total}}}$=${\boldsymbol{m}}_{\mathrm{s}}^{{\mathrm{eff}}}$+***m***_**o**_. The error bars are based on the standard deviation of the Lorentzian-fitting residual

In this section, we first investigate the role of the induced magnetic moment in the Pt layer as mentioned in previous section. The temperature dependence of the Pt-induced magnetic moment was measured using the XMCD method, which enables element-specific analyses of spin and orbital magnetism^33,34^. Figure 3a presents the XMCD and integration of XMCD spectra measured at the Pt *L*_2,3_ edge. The intensities of XMCD are ~3% of the X-ray absorption spectra (XAS) edge heights. At both the *L*_3_ and *L*_2_ edges (around 11.57 keV and 13.28 keV, respectively), the integrated XMCD spectra show temperature dependences. In contrast, there is no temperature dependence in XAS spectra (see inset of Fig. 3a). The total magnetic moment (***m***_total_), which is the sum of an effective spin magnetic moment $\left( {{\boldsymbol{m}}_{\mathrm{s}}^{{\mathrm{eff}}} = {\boldsymbol{m}}_{\mathrm{s}} + {\boldsymbol{m}}_{\mathrm{D}}} \right)$ and ***m***_o_, was estimated by a sum rule calculation^35^ (see the details about the sum rule calculation in Supplementary Note 4). In this study, we use moment values per single hole rather than those per atom because it is difficult to precisely determine the hole number (*n*_h_) of Pt in the Pt/Co/MgO. The moment values normalized by *n*_h_ are directly obtained from the sum rule formula without considering *n*_h_^35^. The changes in the induced magnetic moments with temperature are small (~15%) and comparable to the error range of the analysis as shown in Fig. 3b. This suggests that there is a weak correlation between the temperature dependences of the proximity-induced magnetic moments of the Pt layer and the DMI in the Pt/Co/MgO system^11^.

### Correlation between asymmetric orbital structure of the FM layer and the DMI

In this section, we study the correlation of DMI with various physical quantities associated with the magnetism of Co such as ***m***_s_, ${\boldsymbol{m}}_{\mathrm{o}}^\parallel$ and ${\boldsymbol{m}}_{\mathrm{o}}^ \bot$ and the intra-atomic dipole moment ***m***_D_. As explained in the previous section, ***m***_s,eff_ is the sum of ***m***_s_ and ***m***_D_. The ***m***_s_ and ***m***_D_ values in terms of temperature were obtained from the relation ${\boldsymbol{m}}_{\mathrm{D}}\left( \theta \right) = {\boldsymbol{m}}_{\mathrm{D}}(0^\circ ) \cdot (1 - 3{\mathrm{cos}}^2\theta )$, thereby ${\boldsymbol{m}}_{{\mathrm{s}},{\mathrm{eff}}}\left( \theta \right) = {\boldsymbol{m}}_{\mathrm{s}} + {\boldsymbol{m}}_{\mathrm{D}}(0^\circ ) \cdot (1 - 3{\mathrm{cos}}^2\theta )$^28,34^.

**Fig. 4:**
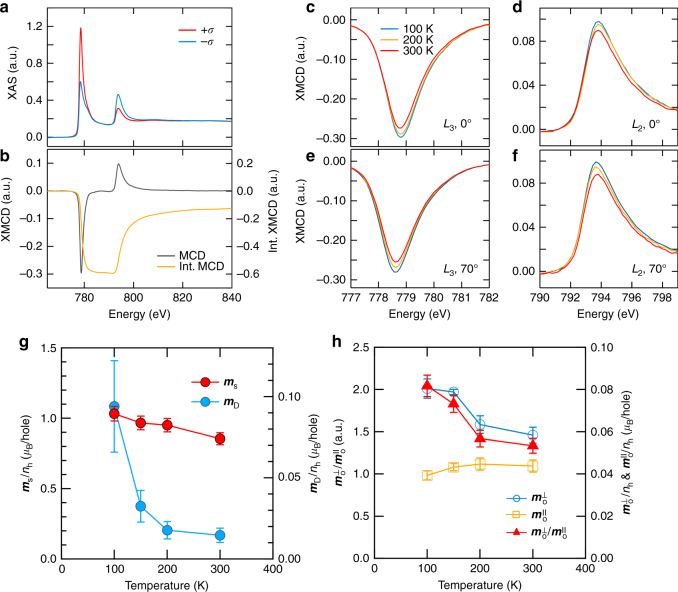
Temperature dependence of the Co magnetic moments. **a** XAS spectra for positive (*σ*^+^) and negative (*σ*−) X-ray helicities. **b** XMCD and integrated XMCD spectra at 0° with 300 K. The temperature dependence of XMCD spectra at the Co *L*_3_ and *L*_2_ edges measured at (**c**, **d**) 0° and (**e**,** f**) 70°. **g** Plots of ***m***_**s**_ and ***m***_D_ vs temperature as a function of the X-ray incident angle. **h** ${\boldsymbol{m}}_{\mathrm{o}}^ \bot$, ${\boldsymbol{m}}_{\mathrm{o}}^\parallel$ and ${\boldsymbol{m}}_{\mathrm{o}}^ \bot /{\boldsymbol{m}}_{\mathrm{o}}^\parallel$ as functions of temperature. The error bars are based on the standard deviation of the integral distribution after subtracting the backgrounds of XAS spectra

Details about the sum rule calculation are explained in Supplementary Note 4. The XAS and XMCD of the film are measured at 100, 200 and 300 K. Two incident angles (*θ*=0° and 70° with respect to the film normal) were used to separately estimate ${\boldsymbol{m}}_{\mathrm{o}}^\parallel$ and ${\boldsymbol{m}}_{\mathrm{o}}^ \bot$ values using the relation ${\boldsymbol{m}}_{\mathrm{o}}\left( \theta \right) = {\boldsymbol{m}}_{\mathrm{o}}^ \bot {\mathrm{cos}}^2\theta + {\boldsymbol{m}}_{\mathrm{o}}^\parallel {\mathrm{sin}}^2\theta$^36^. Figure 4a, b are typical XAS and XMCD spectra at the Co *L*_2,3_ edges obtained at 0° and 300 K. The XMCD spectra show a clear temperature dependence of their intensity at both 0° and 70° as shown in Fig. 4c–f. In case of the ***m***_s_, it increases by 20% (from 0.87 *μ*_B_/*μ*_h_ to 1.11 *μ*_B_/*n*_h_**)** as temperature decreases from 300 to 100 K (see Fig. 4g), which is consistent with the observed temperature dependence of ***M***_s_.

**Fig. 5:**
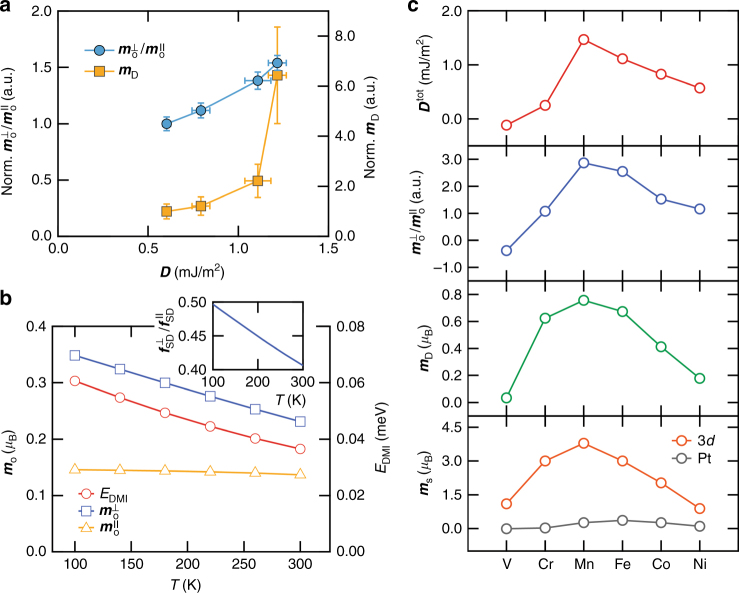
Correlation of DMI with ${\boldsymbol{m}}_{\mathbf{o}}^ \bot /{\boldsymbol{m}}_{\mathbf{o}}^\parallel$ and ***m***_**D**_, and theoretical calculations based on the tight binding model and DFT. **a** Normalized ${\boldsymbol{m}}_{\mathrm{o}}^ \bot /{\boldsymbol{m}}_{\mathbf{o}}^\parallel$ and *m*_D_ vs ***D***. Values of ${\boldsymbol{m}}_{\mathrm{o}}^ \bot /{\boldsymbol{m}}_{\mathbf{o}}^\parallel$ and ***m***_D_ for all temperatures are normalized by the values measured at 300 K. **b** Calculated ${\boldsymbol{m}}_{\mathrm{o}}^ \bot$, ${\boldsymbol{m}}_{\mathbf{o}}^\parallel$ and *E*_DMI_ values based on the tight-binding model as a function of temperature. Inset shows $f_{{\mathrm{SD}}}^ \bot$/$f_{{\mathrm{SD}}}^\parallel$ as a function of temperature. **c** Physical parameters such as total ***D*** (***D***^tot^), ${\boldsymbol{m}}_{\mathrm{o}}^ \bot /{\boldsymbol{m}}_{\mathbf{o}}^\parallel$, ***m***_D_ and ***m***_s_ obtained by DFT calculation. Strength and sign of ***D***^tot^ are calculated around their magnetic ground state using the combination of the relativistic effect spin–orbit coupling with the spin spirals. A positive sign of ***D***^tot^ indicates a left-rotational sense or left chirality

In addition to ***m***_s_, ***m***_D_ shows a strong temperature dependence. The ***m***_D_ reflects the anisotropy of *f*_SD_ distorted by the SOC or the crystal-field effect.^28,29^. When the electron occupation becomes asymmetric, for instance, more electrons in the in-plane orbitals than the out-of-plane orbitals, the situation gives rise to both non-zero ***m***_D_ term and asymmetry in the charge distribution. As a result, asymmetry of the charge distribution naturally implies non-vanishing *f*_SD_ at transition metals' interfaces. As a result, the value of ***m***_D_ determined from the sum rule analysis increases from 0.014 *μ*_B_/*n*_h_ to 0.094 *μ*_B_/*n*_h_ (Fig. 4g). In addition, the orbital anisotropy also increases as temperature decreases; ${\boldsymbol{m}}_o^ \bot$ increases from 0.058 *μ*_B_/*n*_h_ to 0.080*μ*_B_/*n*_h_, whereas ${\boldsymbol{m}}_{\mathrm{o}}^\parallel$ slightly decreases from 0.044 *μ*_B_/*n*_h_ to 0.039 *μ*_B_/*n*_h_ (Fig. 4h). These results imply a correlation of DMI with ***m***_D_ and orbital anisotropy. Since the variation of the perpendicular component of the *m*_o_ is also related to magnetocrystalline anisotropy (***K***_U_) based on the Bruno theory^37^, the correlation between DMI and ***K***_U_ is also reasonable (see the Supplementary Note 5). Figure 5a shows ${\boldsymbol{m}}_{\mathrm{o}}^ \bot /{\boldsymbol{m}}_{\mathrm{o}}^\parallel$(=[${\boldsymbol{m}}_{\mathrm{o}}^ \bot (T)/{\boldsymbol{m}}_{\mathrm{o}}^\parallel (T)$]/[${\boldsymbol{m}}_{\mathrm{o}}^ \bot (300{\mathrm{K}})/{\boldsymbol{m}}_{\mathrm{o}}^\parallel (300{\mathrm{K}})$]), and ${\boldsymbol{m}}_{\mathrm{D}}(T)/{\boldsymbol{m}}_{\mathrm{D}}(300{\mathrm{K}})$ plots as a function of the normalized ***D***=***D***=(*T*)/***D***(300K) where *T* inside of parentheses is the measurement temperature. The ratio ${\boldsymbol{m}}_{\mathrm{o}}^ \bot /{\boldsymbol{m}}_{\mathrm{o}}^\parallel$ increases by 53% as ***D*** increases, and ***m***_D_ also shows a clear correlation with the DMI; ${\boldsymbol{m}}_{\mathrm{D}}(100{\mathrm{K}})/{\boldsymbol{m}}_{\mathrm{D}}(300{\mathrm{K}})$≈ 6.4. Because both orbital anisotropy and ***m***_D_ are closely related to the orbital occupation with ISB^28,37,38^, these results suggest that the temperature dependence of DMI is governed by the change in asymmetric electron occupation in orbitals in addition to spin magnetic moments as we discuss with the following theoretical studies. We also note that peak intensity of the XAS spectra reflects the hole number in 3*d* orbitals. We found small change in the absorption intensity when varying the temperature. Quantitatively, difference in the XAS intensity integral (*I*_XAS_) between 300 and 100 K is about 4% $\{ [I_{{\mathrm{XAS}}}\left( {300{\mathrm{K}}} \right) - I_{{\mathrm{XAS}}}\left( {100{\mathrm{K}}} \right)]/ I_{{\mathrm{XAS}}}\left( {300{\mathrm{K}}} \right) = 0.04\}$. Hence, this indicates that there is a small change in the hole number within the temperature range, reflecting temperature dependence of the charge distribution in orbitals. Nonetheless, the value is so small that it does not affect the trend in the respective quantities normalized by *n*_h_ from the sum rule calculation.

### Theoretical consideration about the microscopic origin of the DMI

In order to support the insights obtained from our experimental study, we carry out two types of theoretical calculations: a tight-binding model calculation and ab initio calculation based on density function theory (DFT), and the main result is shown in Fig. 5b, c. The details of these two complementary theoretical studies can be found in Supplementary Notes 6 and 7.

For the tight-binding model, we extend the trimer model suggested by Kashid et al.^10^, which contains two magnetic atoms coupled to a spin–orbit coupled non-magnetic ion. Compared to Kashid’s trimer model, we add one more orbital (*d*_*xz*_) on the non-magnetic site, enabling the computation of the orbital anisotropy (see details in Supplementary Note 6). In order to describe the temperature dependence of parameters in our trimer model, we assume the level broadening increases with the temperature. This broadening can be phenomenologically explained by magnetization fluctuations and the electron–phonon interaction given by atomic vibrations. As this model calculation is too simple, we do not aim to give a quantitative explanation of the experimental data but provide a qualitative understanding of the experimental result.

Figure 5b shows that the tight-binding model calculation reproduces qualitatively our experimental observations; both the DMI and ${\boldsymbol{m}}_{\mathrm{o}}^ \bot$ decrease with temperature, while the ${\boldsymbol{m}}_{\mathrm{o}}^\parallel$ is almost temperature independent. Note that the ${\boldsymbol{m}}_{\mathrm{o}}^ \bot$ and ${\boldsymbol{m}}_{\mathrm{o}}^\parallel$ cases are related to electron hopping with and without ISB, respectively (Supplementary Note 6). In addition, the ratio of the spin density distribution ($f_{{\mathrm{SD}}}^ \bot$/$f_{{\mathrm{SD}}}^\parallel$) between the in-plane (*xy*) and out-of-plane (*yz*,* xz*) orbital states decreases as temperature increases as shown in the inset in Fig. 5b (In our tight-binding model, the spin distribution is defined as *SD*_*n*_=*f*_*n*,↑_−*f*_*n*,↓_, where *f*_*n*,↑_(*f*_*n*,↓_) is the occupation of spin up (down) state and *n* represents the orbital quantum state $d_{xy}^A,d_{xy}^B,d_{xy}^C,d_{yz}^C,d_{xz}^C$.). This result implies that the spin distribution variation of the orbital states with ISB is key to change of the orbital moment and the DMI, which is in line with the following DFT result.

To examine the correlation between the DMI and the orbital anisotropy in realistic structures, we also perform ab initio calculations for Pt(111)/X ultra-thin films, where X is a 3*d* transition metal (X=V, Cr, Mn, Fe, Co, Ni). Based on the experimental observation, the microscopic origin of the correlation between the DMI and the orbital anisotropy involves the impact of the temperature on 3*d*-orbital magnetization and their electron filling. In this respect, it is instructive to vary the 3*d* transition metals on the Pt substrate and examine the general chemical trend. Details about this study are also discussed in Supplementary Note 7. Figure 5c shows the summary of the DFT calculation. Here changing the overlayer results in modification of the relative alignment between 3*d* and 5*d* orbitals and thereby in a modification of the charge distribution: more hybridization results in less asphericity of the charge distribution between the in-plane and the out-of-plane, as reflected by the change in ***m***_D_. Our calculation results provide a physical insight on how the asymmetric charge distribution induces DMI. As we mentioned in the second section, DMI requires three ingredients: (i) SOC, (ii) magnetic exchange, and (iii) symmetry breaking. While the first two aspects are easily quantifiable, the latter is more difficult to apprehend. It has been reported that the larger the asymmetric spin distribution with symmetry breaking and SOC, the larger intra-dipole spin moment^29^. Therefore, our experimental and theoretical studies propose a method to address the symmetry breaking term. Figure 5c explains this aspect explicitly. The Mn 3*d* orbitals and the Pt 5*d* orbitals provide large magnetic exchange and SOC, respectively. In addition, the dipole moment term becomes maximized with the hybridization between Mn 3*d* and Pt 5*d* orbitals. As a result, DMI is maximal. Furthermore, both the DMI and the orbital anisotropy follow the same trend in their signs: ***D***^tot^ and ${\boldsymbol{m}}_{\mathrm{o}}^ \bot /{\boldsymbol{m}}_{\mathrm{o}}^\parallel$ at the Pt/V interface have negative sign, while those of other interfaces have positive sign. We also find the correlation between ***m***_s_ and DMI as confirmed experimentally in this study and in ref. ^32^. In contrast, our calculations do not reproduce the experimental correlation reported above between DMI and the induced moment. This may be because of the difference between the actual film stack (~3 monolayers of Co) and the simulated system (1 monolayer of Co). In fact, the total DMI is dominated by the contribution of the first Co monolayer at the Co/Pt interface, while the Heisenberg exchange, which is a source of the induced moment, is affected by the total number of Co overlayers. This is why our calculation, limited to one Co overlayer, well reproduces the correlation of DMI with spin and orbital moments, but not with the induced moment.

**Fig. 6:**
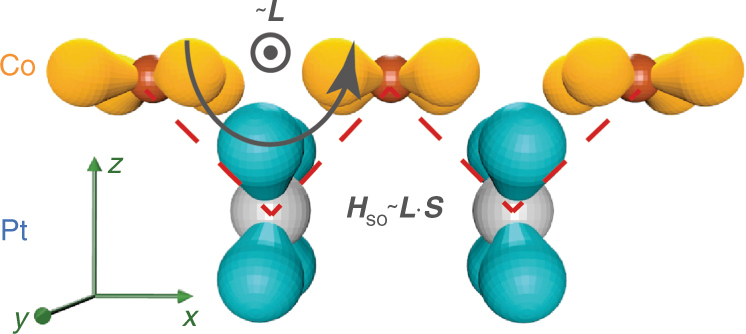
Schematic illustration of the spin canting. Explicit illustration of the onset of the orbital angular momentum and how SOC induces spin canting from it. The red and grey spheres represent the top ferromagnetic and bottom normal metal atoms in each layer, while the yellow and blue spheres represent the *d*_*xy*_ (top) and *d*_*yz*_ (bottom) orbitals, respectively. Dotted red lines and black curved arrow indicate the electron hopping path and effective electron orbiting motion due to the hopping, respectively

Now, the remaining question is how the orbital asphericity can give rise to DMI. At the interface, in-plane orbitals of Co and out-of-plane orbitals of Pt are mixed through inter-atomic hybridization (in our tight-binding model: *d*_*xy*_ of Co and *d*_*yz*_ of Pt). In the presence of ISB, this hybridization results in a non-vanishing orbital angular momentum: the hopping trajectory of an electron produces an effective orbital angular momentum as illustrated in Fig. 6. Moreover, the large SOC on Pt mixes in-plane and out-of-plane orbitals, which is accompanied by spin canting (in our tight-binding model: *d*_*xy*_ and *d*_*yz*_ orbitals on Pt site). In other words, the SOC on Pt converts the interfacial orbital angular momentum of hopping electrons into spin canting, resulting in a non-collinear magnetic texture. In spite of its simplicity, this picture intuitively explains how the orbital asphericity generates the spin canting. A similar idea has been reported in ref. ^21^: the interfacial electric dipole induced by the asymmetric charge distribution results in chiral orbital angular momentum, and thereby spin canting via SOC. Although the latter approach is based on *p*-orbitals, it agrees with the intuitive picture based on *d*-orbitals.

## Discussion

Our experimental study on the temperature dependence of the DMI suggests that the interfacial DMI in FM/HM bilayers originates from Heisenberg exchange and the asymmetric charge distribution caused by the ISB, as evidenced by the orbital anisotropy and magnetic dipole moment (${\boldsymbol{m}}_{\mathrm{o}}^ \bot /{\boldsymbol{m}}_{\mathrm{o}}^\parallel ,$ ***m***_D_). Our DFT simulation and tight-binding calculation provide a clear evidence of the close link between the DMI and orbital physics. Based on the theoretical discussions, the temperature-dependent ***m***_D_ and ***m***_o_ indicate that increase in the temperature promotes the phonon-induced electron hopping between the out-of-plane and in-plane orbitals, thereby resulting in a reduced asphericity (or reduced asymmetry) of *f*_SD_ over the *d*-orbitals and thus quenching ***m***_D_.

In our experiment, however, the correlation between ${\boldsymbol{m}}_{\mathrm{o}}^ \bot /{\boldsymbol{m}}_{\mathrm{o}}^\parallel$ and the DMI is only semi-quantitative, i.e. the temperature-dependent change in the DMI does not perfectly scale with the temperature-dependent change in ${\boldsymbol{m}}_{\mathrm{o}}^ \bot /{\boldsymbol{m}}_{\mathrm{o}}^\parallel$. This semi-quantitative correlation between the DMI and ***m***_o_ demands a more detailed discussion. In systems with ISB, ${\boldsymbol{m}}_{\mathrm{o}}^ \bot$ can be decomposed into ISB-independent part and ISB-dependent part. Given that ISB is an essential ingredient for the DMI, there should be a direct correlation between the DMI and ISB-dependent ***m***_o_, as evidenced by our tight-binding model calculation. However, ${\boldsymbol{m}}_{\mathrm{o}}^ \bot$ also has an ISB-independent part, which precludes a direct and quantitative correlation between the DMI and ${\boldsymbol{m}}_{\mathrm{o}}^ \bot$. This statement can be rephrased technically as the DMI involves only off-diagonal elements of the SOC operator^10^, while ***m***_o_ involves all of them. We note, however, that even with this uncertainty, the experimentally observed correlation between the DMI and ***m***_o_ anisotropy for the Pt/Co/MgO structure is rather clear, implying that the ISB-dependent ***m***_**o**_ would dominate over the ISB-independent one in this structure. Therefore, our findings based on both experimental and theoretical studies provide a link between orbital physics and spin–orbit-related phenomena such as the DMI, which are essential for spin–orbitronic devices.

## Method

### Film preparation and device fabrication

Si/Ta (1.5)/Pt (2)/Co (0.5)/MgO (2)/HfO (5) (in nm) film with perpendicular magnetic anisotropy was deposited on an undoped Si substrate by direct current magnetron sputtering and the atomic layer deposition technique. A 5-μm-wide Hall cross-structure were fabricated using the photo lithography and the Ar ion milling. For the XMCD measurement, the same stack film was prepared.

### Nucleation field measurement

Angular-dependent coercivity of the Co/Pt Hall device was measured to estimate the ***H***_n_ of the magnetic droplet at 300, 200, 150 and 100 K. The angle between magnetic field and the sample normal was varied from 0° to 89° rotating the electromagnet. At each angle, magnetic field was swept with in ±0.5 T to observe the coercivity. Details about this measurement are also discussed in ref. ^24^.

### XMCD measurement

Soft X-ray: Soft X-ray absorption spectra were measured using the total electron yield method with 96% circularly polarized incident X-rays at the BL25SU at SPring-8. XMCDs at the Co *L*_3_ and *L*_2_ edges (in a range between 770 and 840 keV) were recorded in the helicity-switching mode with an applied magnetic field of 1.9 T. Homogeneity of the magnetic field was better than 99% for *ɸ*10 mm at the sample position. The incident light direction was inclined by 10° with respect to the magnetic field direction. Temperature was varied from 300 to 100 K using a continuous liquid He flow-type cryostat.

Hard X-ray: XMCD experiments using hard X-rays were carried out at BL39XU of SPring-8. A circularly polarized X-ray beam with a high degree of circular polarization (>95%) was produced with a transmission-type diamond X-ray phase retarder of 1.4-mm thickness. XAS of the film were observed at a 0.6 T magnetic field applied parallel to the X-ray propagation direction of which an incident angle was 0° with respect to the surface normal. The X-ray fluorescence yield mode was used to record the spectra. The X-ray energy was scanned around the Pt *L*_3_ and *L*_2_ edges in a range between 11.5 and 3.5 keV, reversing the X-ray photon helicity at 0.5 Hz. In this manner, two helicity dependent spectra *I*^+^ and *I*^*−*^ were recorded simultaneously. Here *I*^+^ and *I*^−^ denote the intensities when the incident photon momentum and the magnetization vectors are parallel and antiparallel, respectively. The XMCD spectrum, Δ*I* = *I*^+^−*I*^*−*^, is given by the difference of the two spectra. Detailed experimental set-ups for the soft and hard X-ray MCD measurements are described elsewhere^39,40^.

### DFT calculation

To understand the behaviour of DMI in 3*d*/Pt(111) ultrathin films and its correlation with the orbital moment anisotropy (OMA) and magnetic dipole moment (*T*_z_), we have performed DFT calculations in the local density approximation^41^ to the exchange correlation functional, using the full potential linearized augmented plane wave method in film geometry^42^ as implemented in the Jülich DFT code FLEUR^43^. Both collinear and non-collinear magnetic states have been studied employing an asymmetric film consisting of six substrate layers of Pt covered by a pseudomorphic 3*d* monolayer on one side of the film at the distance optimized for the lowest collinear magnetic states. For the non-collinear calculations, we used *p*(1x1) unit cell applying the generalized Bloch theorem (http://www.flapw.de). The OMA is obtained at relaxed geometry as total energy difference for two different magnetization directions employing the force theorem (the principle axes point along hard and easy axis). We considered 512 and 1024 *k*-points in the two-dimensional Brillouin zone (2D-BZ) for the scalar relativistic computation and the calculation with SOC treated within first-order perturbation theory, respectively. Note that high computational accuracy is required since energy differences between different magnetic configurations are tiny (~meV) in the present case.

In order to investigate the DMI, first we self-consistently calculate the total energy of homogeneous magnetic spin spirals employing the generalized Bloch theorem within the scalar-relativistic approach^44^. We have considered the energy dispersion *E*(q) of planar spin spirals, which are the general solution of the Heisenberg Hamiltonian, i.e. states in which the magnetic moment of an atom site *R*_*i*_ is given by *M*_*i*_=*M*[cos(*q*·*R*_*i*_), sin(*q*·*R*_*i*_), 0] where *q* is the wave vector propagation of the spin spiral. By imposing the Néel spin spirals along the high symmetry lines of 2D-BZ, we can scan all possible magnetic configurations that can be described by a single *q*-vector. So, varying the *q*-vector with small steps along the paths connecting the high symmetry points, we find the well-defined magnetic phases of the hexagonal lattice: FM state at ${\bar{\mathrm \Gamma }}$-point (*q*=0), anti-FM state at the ${\bar{\mathrm M}}$-point, and periodic 120° Néel state the ${\bar{\mathrm K}}$-point. When the energy *E*(*q*) along the high symmetry lines of 2D-BZ is lower than any of the collinear magnetic phases studied previously, the system most likely adopts an incommensurate spin-spiral magnetic ground state structure.

In a second step, we evaluate the DMI contribution from the energy dispersion of spin spirals by applying the SOC treated within first-order perturbation theory combined with the spin spirals^5,42^. Phenomenologically, the antisymmetric exchange interaction DMI has the typical form $E_{{\mathrm{DM}}} = \mathop {\sum }\limits_{i,j} {\boldsymbol{D}}_{i,j} \cdot ({\boldsymbol{S}}_i \times {\boldsymbol{S}}_j)$, where ***D***_*i*__,__*j*_ is the DM vector that determine the strength and sign of DMI and ***S***_*i*_ and ***S***_*j*_ are magnetic spin moments located on neighbouring atomic sites *i* and *j*, respectively. Considering the Néel-type out-of-plane configuration, the ***D***_*i*__,__*j*_ vector should be oriented in plane and normal to the *q*-vector. Note that DMI term must vanish for both configurations Néel-type in-plane and Bloch-type spin spirals due to symmetry arguments^44^. According to our definition, the vector chirality reads $\boldsymbol{C}= C\hat{\boldsymbol c} = {\boldsymbol{S}}_i \times {\boldsymbol{S}}_{i + 1}$, where the direction of the vector spin chirality $\hat{\boldsymbol c}$ is considered as spin rotation axis. Thus the left-handed (right-handed) spin spiral correspond to *C* = +1 (*C* = −1)^45^.

### Data availability

The data that support the findings of this study are available from the corresponding author upon reasonable request.

## Electronic supplementary material

Supplementary Notes
