# Supplementary material for: Correlation of the Dzyaloshinskii–Moriya interaction with Heisenberg exchange and orbital asphericity
Source: Nat Commun. 2018 Apr 25;9:1648. doi: 10.1038/s41467-018-04017-x (PMC5916936; doi:10.1038/s41467-018-04017-x)
Supplement: Supplementary file 1 — Supplementary Notes [file 41467_2018_4017_MOESM1_ESM.pdf]

Supplementary Note 1.

Domain wall energy calculation with the extended Droplet model

Nucleation field ($\boldsymbol{H}_{\mathrm{n}}$) of a magnetic droplet (as illustrated in Supplementary Figures 1a and 1b) is a quadratic function of the DW energy (σ_{DW}); $H_{\mathrm{n}} \propto \sigma_{\mathrm{DW}}^2$ [1-3]. The σ_{DW} is generally given by [2]

$$\sigma_{\mathrm{DW}} = \begin{cases} \sigma_0 - \dfrac{\pi^2 \Delta \boldsymbol{M}_{\mathrm{S}}{}^2 \mu_0{}^2}{8K_{\mathrm{D}}} (\boldsymbol{H}_x + \boldsymbol{H}_{\mathrm{DMI}})^2 & , \text{ for } \mu_0 |\boldsymbol{H}_{\mathrm{x}} + \boldsymbol{H}_{\mathrm{DMI}}| < \dfrac{4K_{\mathrm{D}}}{\pi \boldsymbol{M}_{\mathrm{S}}} \\ \sigma_0 + 2K_{\mathrm{D}}\Delta - \pi \Delta \boldsymbol{M}_{\mathrm{S}} \mu_0 |\boldsymbol{H}_X + \boldsymbol{H}_{\mathrm{DMI}}| & \text{otherwise,} \end{cases} \quad (1)$$

where σ_0 (=$4\sqrt{AK_{\mathrm{eff}}}$) is the DW energy with Bloch-type, A is the exchange stiffness constant, K_{eff} is the effective perpendicular anisotropy energy, and Δ (=$\sqrt{A/K_{\mathrm{eff}}}$) is the domain wall width. $4K_{\mathrm{D}}/\pi\boldsymbol{M}_{\mathrm{S}}$ is the magnetic field magnitude required to saturate the magnetization in the domain wall of droplet with H_{x} (K_{D} is DW anisotropy energy). Because σ_{DW} is influenced by DMI and $\boldsymbol{H}_x$ [2], it is expected that DMI energy density ($\boldsymbol{D}$) can be estimated from the measurement of H_{n} as a function of $\boldsymbol{H}_x$. Based on our extended droplet model [4], $\boldsymbol{H}_{\mathrm{DMI}}$ under $\boldsymbol{H}_x$ can be estimated from the relation between $\boldsymbol{H}_{\mathrm{n}}$ and σ_{DW}. When D is stronger than K_{D}, particularly the case of Pt/Co structures, DW magnetizations are aligned in the radial direction. In the extended droplet model [4], only two DW magnetizations with respect to H_x is considered as shown in Supplementary Figure 1b with white arrows. Total DW energy under H_x can be given by $\sigma_{DW,total}(|\boldsymbol{H}_x|) = \sigma_{\mathrm{DW1}}(+\boldsymbol{H}_x) + \sigma_{\mathrm{DW2}}(-\boldsymbol{H}_x)$ because magnetizations of two DW sections aligned in the x-direction are approximately anti-parallel with each other. The ± sign indicates a DW magnetization with the parallel (+) or antiparallel (-) alignment to $\boldsymbol{H}_x$. When $|\boldsymbol{H}_x|$ ~0 mT, the $\sigma_{\mathrm{DW,total}}$ is reduced by only $\boldsymbol{H}_{\mathrm{DMI}}$. However, when $|\boldsymbol{H}_x|>|\boldsymbol{H}_{\mathrm{DMI}}|$, $\sigma_{\mathrm{DW,total}}$ is not influenced by $\boldsymbol{H}_{\mathrm{DMI}}$, then given by

$$\sigma_{\mathrm{DW,total}}(|\boldsymbol{H}_x|) = 2\sigma_0 + 4K_{\mathrm{D}}\Delta - 2\pi\Delta\boldsymbol{M}_{\mathrm{S}}\mu_0\boldsymbol{H}_x. \quad (2)$$

We map $\sigma_{\mathrm{DW,total}}{}^2$ of a Pt/Co/MgO trilayer in terms of $\boldsymbol{H}_{\mathrm{DMI}}$ and $\boldsymbol{H}_x$ based on this extended droplet model as shown in Supplementary Figure 1c. Note that there is threshold $\boldsymbol{H}_x$ where $\boldsymbol{H}_x$ becomes effective on $\sigma_{\mathrm{DW,total}}{}^2$ (thus $\boldsymbol{H}_{\mathrm{n}}$) as marked with the white dotted line in Supplemen tary Figure 1c. The threshold $\boldsymbol{H}_{\mathrm{x}}$ linearly increases with $\boldsymbol{H}_{\mathrm{DMI}}$ (see also Supplementary Figure 1d). Therefore, $\boldsymbol{H}_{\mathrm{DMI}}$ can be estimated by measuring a threshold $\boldsymbol{H}_x$ [4].

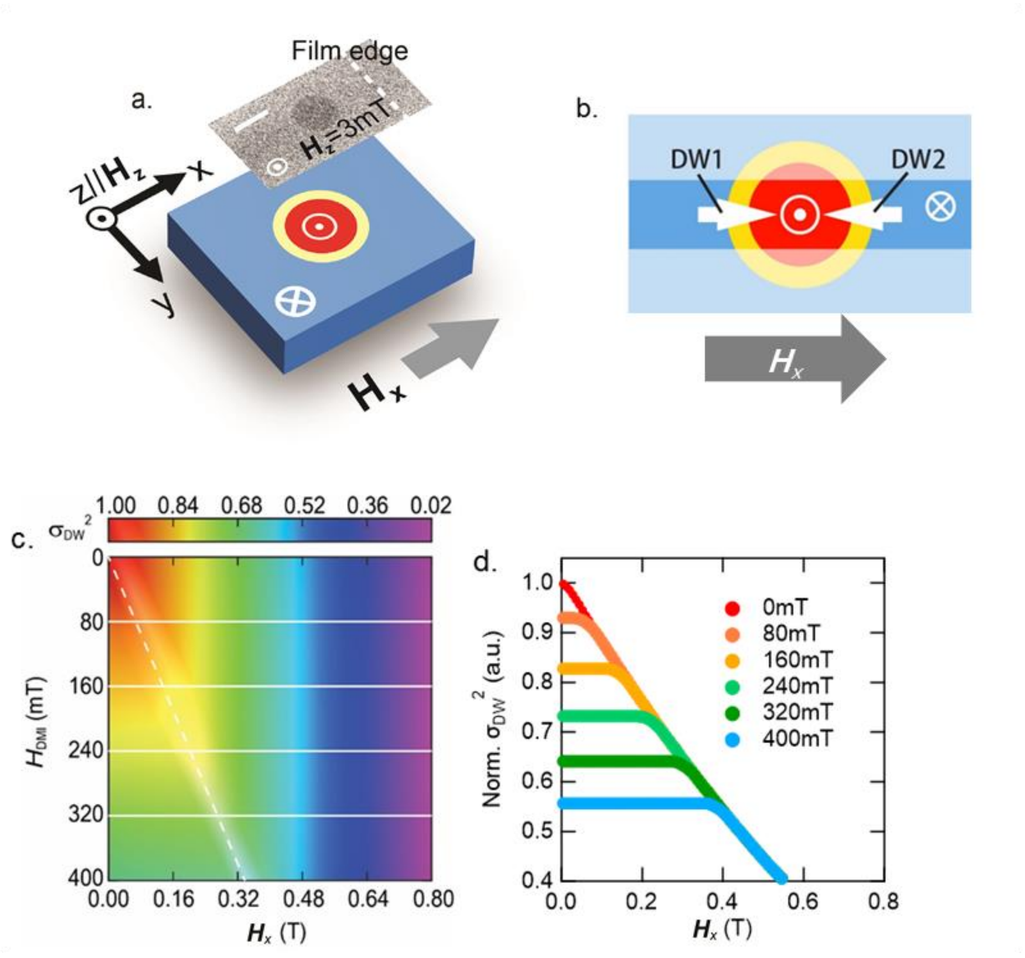

Supplementary Figure 1. Droplet model to estimate DMI. Schematic images of **a.** a magnetic droplet in a ferromagnetic medium and **b.** Two DW configurations which are antiparallel to each other. The inset is the MOKE image which displays the droplet nucleates in the film rather than the edge. The white bar is the scale bar of 5 μm. The grey arrow illustrates the $\boldsymbol{H}_x$. The red circle in the blue medium is the droplet with magnetization-up state. The yellow boundary of the droplet is the DW, and the white arrows are the magnetization of the DW. **c.** DW energy density mapping in terms of $\boldsymbol{H}_{\mathrm{DMI}}$ and $\boldsymbol{H}_x$. The white dotted line is guide for an eye indicating the threshold $\boldsymbol{H}_x$ for the DW energy drop by $\boldsymbol{H}_x$. White solid grids are also guide for an eye. For this calculation, we used the parameters obtained from data at 300K as listed in Table 1 in the main manuscript. **d.** Normalized $\sigma_{\mathrm{DW}}{}^2$ vs $\boldsymbol{H}_x$ curves with the selected $\boldsymbol{H}_{\mathrm{DMI}}$ values.

Supplementary Note 2.

Determination of DMI based on the string model

In order to determine the DMI strength from experimental results, we calculate the energy barrier E_B as a function of the in-plane field $\boldsymbol{H}_x$ and out-of-plane field $\boldsymbol{H}_z$, based on the string method [5]. Details about the string method are explained in Ref. 5~6. In Supplementary Figure 2, we show simulation results at three temperatures (T = 100, 200, and 300 K), using the parameters experimentally measured for each temperature (see Supplementary Table 1). In these plots, we normalize the horizontal axis with the effective perpendicular anisotropy field H_K and the vertical axis with H_{z0} which is the out-of-plane field having the same E_B as in experiments for $H_x = 0$. By comparing these numerical results with experimental ones, we determine the DMI values: $\boldsymbol{D}$ = 1.3~1.5 mJ·m^{-2} at T = 100 K, $\boldsymbol{D}$ = 0.7~0.9 mJ·m^{-2} at T = 200 K, and $\boldsymbol{D}$ = 0.4~0.6 mJ·m^{-2} at T = 300 K.

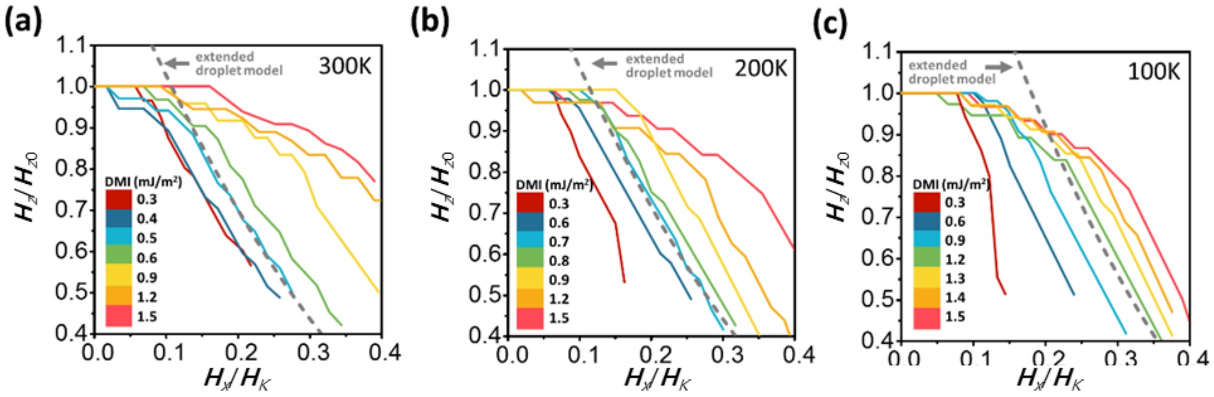

Supplementary Figure 2. Micromagnetic simulation results. E_B boundaries for various DMI constants at **a,** temperature T = 300 K, **b,** T = 200 K, and **c,** T = 100 K. Gray dotted lines are obtained from the extended droplet model.

Supplementary Table 1. Parameters for the simulation and the droplet model calculation. Regarding the temperature dependence of *A*, we estimated *A* values as explained in the section 3 in this Supplementary Information, in which the authors found that *A* is proportional to M_s^n (n≤2) below the Curie temperature.

Temperature (K)	100	200	300
t (nm)	0.4		
A (J/m)	9.22×10^{-12}	7.26×10^{-12}	5.85×10^{-12}
P	69	34	23
$\boldsymbol{H}_K$ (T)	2.24	1.99	1.45
$\mu_0\boldsymbol{M}_\mathrm{S}$ ($\mathrm{J\cdot T^{-1}\cdot m^{-3}}$)	1.33×10^{6}	1.22×10^{6}	1.06×10^{6}
Measured $\boldsymbol{H}_\mathrm{DMI}$ (T)	0.37	0.25	0.17
$\boldsymbol{D}$ ($\mathrm{mJ\cdot m^{-2}}$) from measured $\boldsymbol{H}_\mathrm{DMI}$	0.77	0.52	0.35
$\boldsymbol{D}$ ($\mathrm{mJ\cdot m^{-2}}$) from the simulation	1.3~1.5	0.7~0.9	0.4~0.6

Supplementary Note 3.

Determination of the exchange stiffness constant A

We estimate the temperature dependence of A from the $\boldsymbol{M}_s$-T curve as follows; firstly, we get the spin wave stiffness D_{spin} from fitting with the Bloch $T^{3/2}$ law (eq. 1) as shown in Supplementary Figure 3;

$$\frac{\boldsymbol{m}_s(T)}{\boldsymbol{m}_s(0)} = 1 - \frac{g\mu_B\eta}{\boldsymbol{m}_s(0)} \cdot \left(\frac{k_B T}{\boldsymbol{D}_{spin}(0)}\right)^{\frac{3}{2}}, \tag{1}$$

where g is Landé g-factor, $\boldsymbol{m}_s(0)$ is the saturation magnetization at 0 K, μ_B is Bohr magneton, k_B is Boltzmann constant, and η is a dimensionless constant which depends on sample dimensions. Then, the exchange stiffness A at 0 K, $A(0)$, was estimated using the relation $A(0) = \boldsymbol{D}_{spin}(0) \cdot \boldsymbol{m}_s(0\,)/g\mu_B$ [7]; $A(0)$ and η are 10.2 pJ/m and 0.11, respectively. Finally, the exchange stiffness at each temperature, $A(T)$ was calculated using the relation;

$$\frac{A(T)}{A(0)} \cong \left[\frac{\boldsymbol{M}(T)}{\boldsymbol{M}_s(0)}\right]^{\gamma} \cdot \left[\frac{\boldsymbol{M}(T)}{\boldsymbol{M}_s(0)}\right]. \tag{2}$$

Because γ=1 based on the mean field theory, Eq. (2) can be simplified as

$$\frac{A(T)}{A(0)} \cong \left[\frac{\boldsymbol{M}(T)}{\boldsymbol{M}_s(0)}\right]^{2}. \tag{3}$$

Error was determined, considering that γ can be in the range from 4/3~0.7 [7]. The estimated values are listed in Table 1 in the manuscript. DMI energy constant $\boldsymbol{D}$ values are obtained applying the A values for the droplet model fitting.

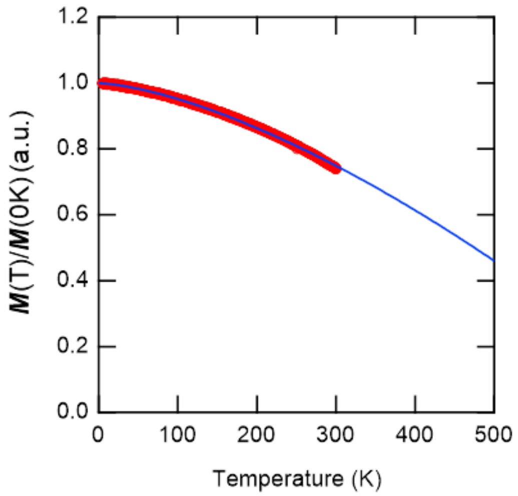

Supplementary Figure 3. Temperature dependence of the $\boldsymbol{M}_s$. $\boldsymbol{M}(T)/\boldsymbol{M}$(0K) vs. T curve**.**

Supplementary Note 4.

Determination of the magnetic anisotropy energy K_u

Perpendicular magnetic anisotropies (PMA) of the Pt/Co/MgO trilayer were estimated by the area method using the hard axis *M-H* curve at each temperature as shown below in Supplementary Figures 4 a-c. *M* and *H* are magnetic moment and magnetic field, respectively. Since the remanent magnetization is the same as M_s, the marked area with red in the figures indicate PMA energy density (K_U). As temperature decreases from 300K to 100K, PMA increases by a factor of two (from 8.27×10^5 J/m^3 to 1.60×10^6 J/m^3). According to the Bruno theory [8], difference between $\boldsymbol{m}_o^{\perp}$ and $\boldsymbol{m}_o^{\parallel}$ ($\Delta\boldsymbol{m}_o$) is proportional to PMA as shown in Supplementary Figure 5, thereby the correlation between DMI and PMA can be found.

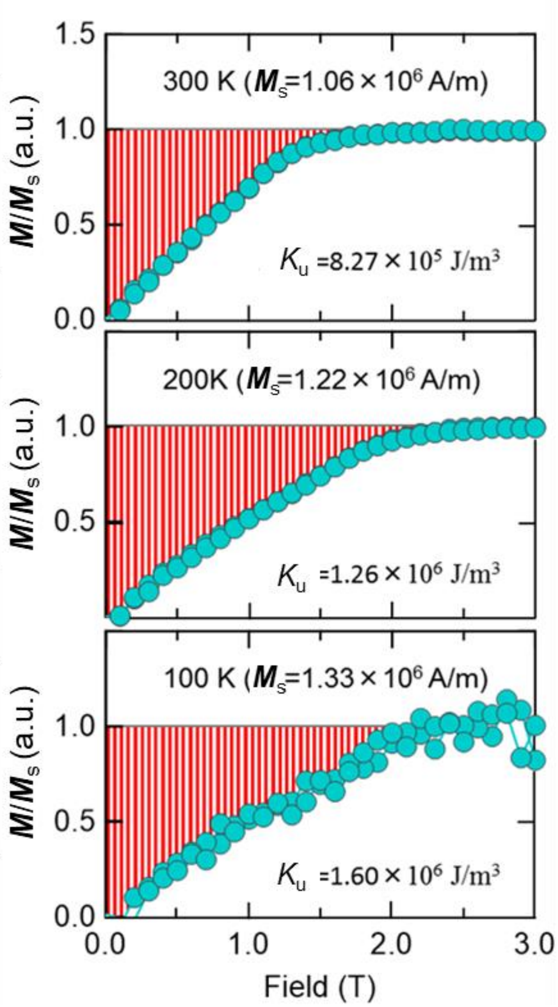

Supplementary Figure 4. Temperature dependence of hard axis magnetization. Hard axis ***M-H*** curves at 100, 200, and 300 K.

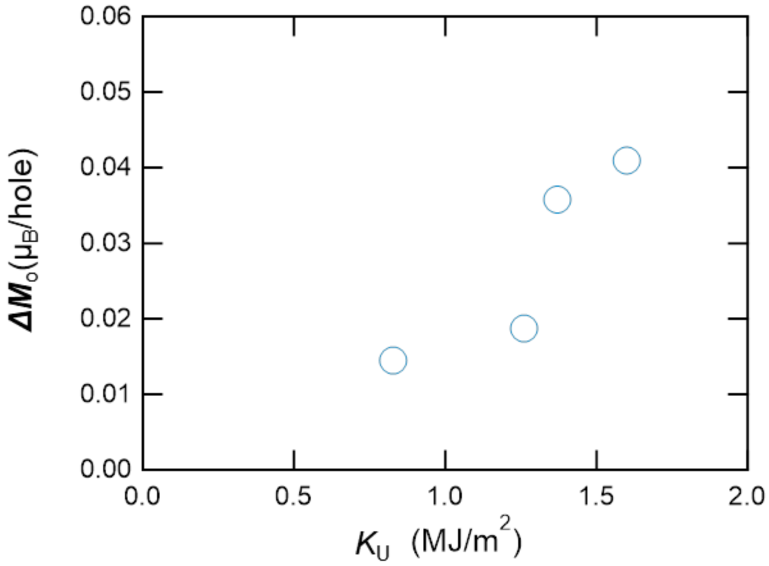

Supplementary Figure 5. Correlation between Δm_o and PMA. Plots of Δm_o in terms of K_u.

Supplementary Note 5.

1. Sum rule analysis

a. *Sum rule analysis for the 3d and 5d transition metals*

According to the sum rule, the orbital magnetic moment $\boldsymbol{m}_o$ and the effective spin magnetic moment $\boldsymbol{m}_s - 7\boldsymbol{m}_T$, where $\boldsymbol{m}_{spin}$ and $\boldsymbol{m}_T$ are respectively the spin and the dipole magnetic moments of a $3d$ and $5d$ transition metal, can be determined as follows [9,10];

$$-\frac{2}{3}\left(\frac{\Delta A_{L_3} + \Delta A_{L_2}}{A_{total}}\right) = \frac{1}{n_h \mu_B}\boldsymbol{m}_o, \text{ and} \tag{4}$$

$$-\left(\frac{\Delta A_{L_3} - 2\Delta A_{L_2}}{A_{total}}\right) = \frac{1}{n_h \mu_B}(\boldsymbol{m}_s - 7\boldsymbol{m}_T), \tag{5}$$

where $A_{total} = \int_{L_3} I(E)dE + \int_{L_2} I(E)dE$ is the XAS integral summed over the L_3 and L_2 edges. $\Delta A_{L_3} = \int_{L_3} \Delta I(E)dE$ and $\Delta A_{L_2} = \int_{L_2} \Delta I(E)dE$ are the integrals of the XMCD spectra at the L_3 and L_2 edges, respectively. $\Delta I = I_+(E) - I_-(E)$. n_h is the hole number of the d band for the transition metals, and μ_B is the Bohr magneton. This sum rule calculation may include ~15% errors from the background elimination, approximation of n_h, and theoretical error of the sum rule itself.

b. *Sum rule calculation for the Co $L_{2,3}$ edge*

Co $L_{2,3}$ edge of the film II was observed in a soft x-ray regime as shown in Supplementary Figure 6 (typical XAS and XMCD spectra are presented in Supplementary Figure 6a). While the XMCD spectra in Fig. 3 of the main manuscript present temperature dependence, XAS does not show temperature dependence. Note that the background of XAS spectra at 810 eV slightly decreases at 100 and 150K. However, the A_{total} values are almost the same as those measured at each temperature. As mentioned above, this background treatment may give some error for this sum rule analysis. For the sum rule calculation, n_h=2.40 was assumed from the 1st principle calculation [9-12]. n_h, which is sensitive to an atomic environment, is required to be carefully determined for the reliable results. Here, since temperature is a sole term we dealt with, there is no significant chemical or crystalline modifications at the interface. Therefore, we believe that this n_h does not affect the trend of the calculation result. A_{total} was also determined as an area of the white line above an edge-jump background (the arctangent step function) in the XAS spectrum. $\boldsymbol{m}_o/\boldsymbol{m}_s^{eff}$, m_o, and m_s were calculated using the sum rule. Out-of-plane ($\boldsymbol{m}_o^{\perp}$) and in-plane ($\boldsymbol{m}_o^{\parallel}$) orbital magnetic moments were estimated by the least-square fitting of the measured moment values at θ = 0, 55, and 70° using the relation: $\boldsymbol{m}_o = \boldsymbol{m}_o^{\parallel}\sin^2\theta + \boldsymbol{m}_o^{\perp}\cos^2\theta$.

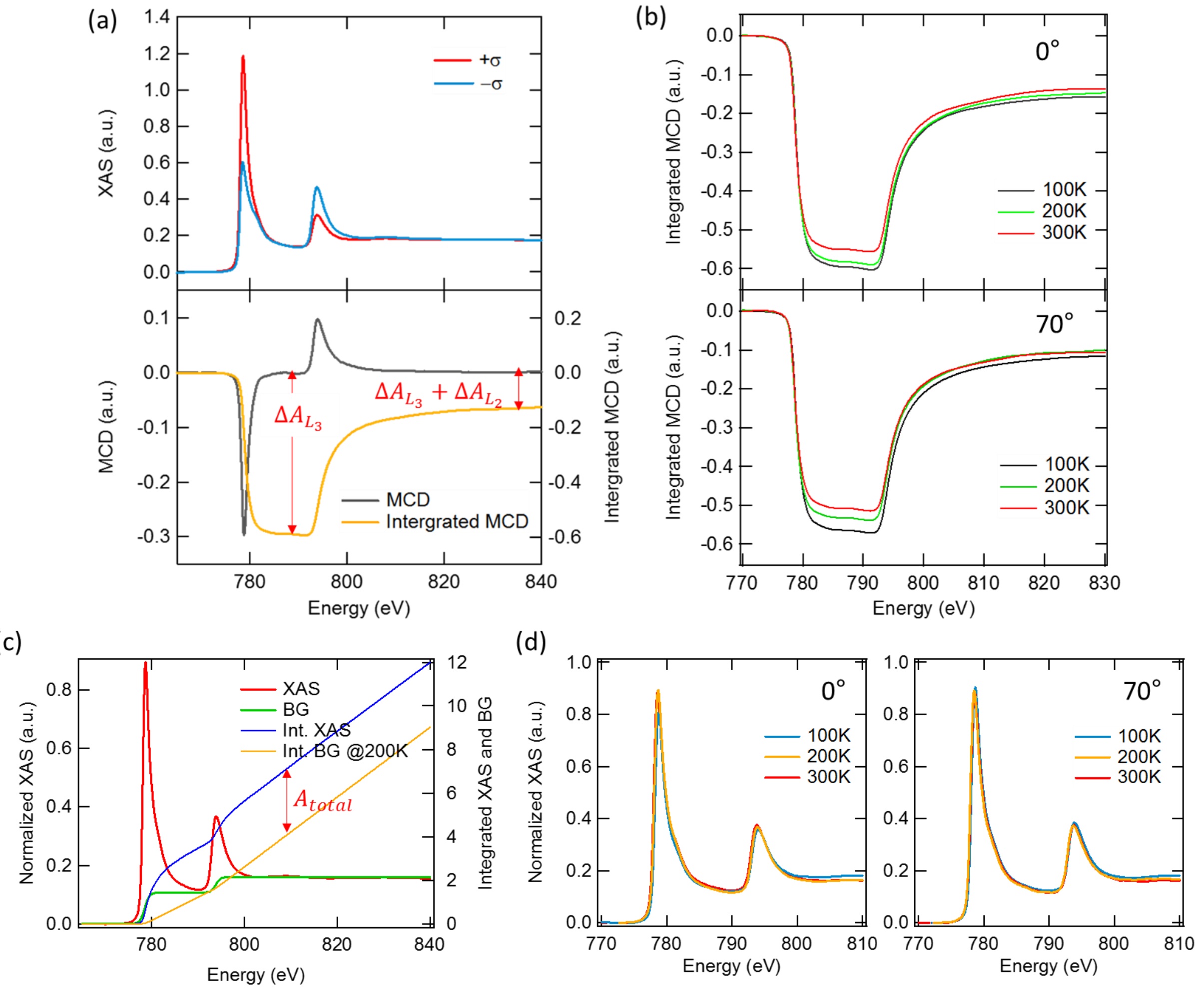

Supplementary Figure 6. XMCD measurement results. a. (top) XAS spectra with positive (σ+) and negative (σ-) helicities of x-ray, and (bottom) XMCD and integrated XMCD spectra of Co *L* edge. Those were measured at 200K, 0°. **b.** The integrated XMCD spectra measured at (top) 0° and (bottom) 70° in terms of temperature. **c.** Example of the normalized XAS (200K, 0°). BG is the abbreviation of a 'background'. BG is fitted line using the arctangent step function. **d.** The XAS spectra measured with (left) 0° and (right) 70° in terms of temperature.

Supplementary Note 6.

Correlation between orbital moment and Dzyaloshinskii-Moriya interaction in a trimer model

a. Model Hamiltonian and calculation scheme

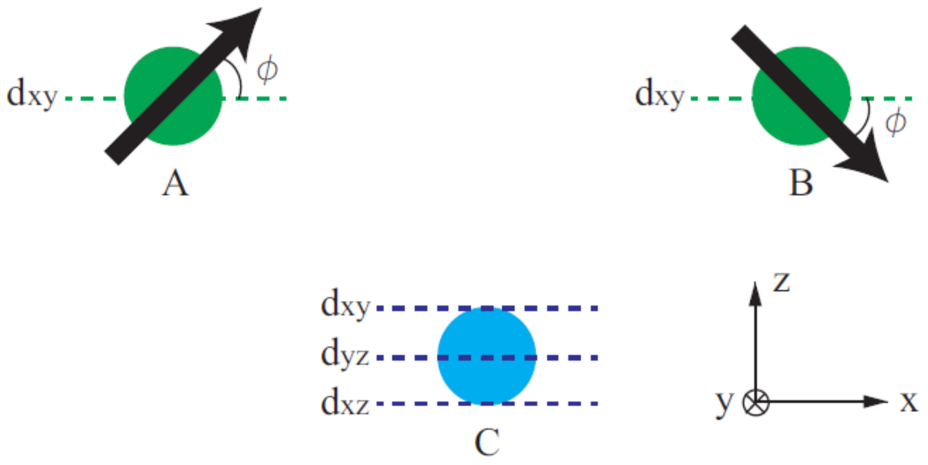

Supplementary Figure 7. Selected orbitals for the trimer model. Structure of the trimer with two identical magnetic sites (A and B) and one nonmagnetic site (C). ϕ is the angle of magnetic moment relative to x -axis.

As a minimal model, we adopt a tight-binding model that is extended from the model proposed by Kashid *et al* [13]. Our model is based on an atomic trimer with two identical magnetic sites and one nonmagnetic site (Supplementary Figure 7). We consider three energetically degenerate d_{xy}, d_{yz}, and d_{xz} orbitals (t_{2g}) at the nonmagnetic sites and a d_{xy} orbital at each magnetic site (note that the coordinate orientation is different from that of Kashid *et al*). Compared to Kashid's model, we add one more orbital (d_{xz}) on the nonmagnetic site, which allows us to compute the orbital moment anisotropy ($\boldsymbol{L}_x$ and $\boldsymbol{L}_z$). Because the hopping integrals between d_{xz} at nonmagnetic site and d_{xy} at two magnetic sites are zero from the Slater-Koster parameterization, the additional d_{xz} orbital at the nonmagnetic site does not contribute to Dzyaloshinskii-Moriya interaction (DMI) whereas it produces nonzero orbital magnetic moments in the trimer's plane ($\boldsymbol{M}_x^{\mathrm{o}}$ and $\boldsymbol{M}_z^{\mathrm{o}}$). Therefore, this model is the simplest framework to investigate the relation between DMI and orbital moment anisotropy. Our model is too simple to capture a quantitative relation between DMI and orbital moment anisotropy, but may give an insight into a qualitative feature.

In representation of the basis $(d_{xy}^A, d_{xy}^B, d_{xy}^C, d_{yz}^C, d_{xz}^C)$, we have 10-state model (without spin-orbit interaction)

$$H_0 = \left(\begin{array}{ccccc|ccccc}
E_A + Im/2\cos\phi & 0 & t_1 & t_2 & 0 & -Im/2\sin\phi & 0 & 0 & 0 & 0 \\
0 & E_A + Im/2\cos\phi & t_1 & -t_2 & 0 & 0 & Im/2\sin\phi & 0 & 0 & 0 \\
t_1 & t_1 & E_C & 0 & 0 & 0 & 0 & 0 & 0 & 0 \\
t_2 & -t_2 & 0 & E_C & 0 & 0 & 0 & 0 & 0 & 0 \\
0 & 0 & 0 & 0 & E_C & 0 & 0 & 0 & 0 & 0 \\
\hline
-Im/2\sin\phi & 0 & 0 & 0 & 0 & E_A - Im/2\cos\phi & 0 & t_1 & t_2 & 0 \\
0 & Im/2\sin\phi & 0 & 0 & 0 & 0 & E_A - Im/2\cos\phi & t_1 & -t_2 & 0 \\
0 & 0 & 0 & 0 & 0 & t_1 & t_1 & E_C & 0 & 0 \\
0 & 0 & 0 & 0 & 0 & t_2 & -t_2 & 0 & E_C & 0 \\
0 & 0 & 0 & 0 & 0 & 0 & 0 & 0 & 0 & E_C
\end{array}\right), \tag{6}$$

where $E_A(= E_B)$ and E_C are respectively on-site energies of A, B and C atoms, ϕ is the angle of magnetic moment relative to the global spin-quantization axis, I is the Stoner parameter corresponding to the magnetic moment m. t_1 and t_2 are hopping integrals between A, B and C atoms. t_1 is the hopping integral between d_{xy} orbitals at a magnetic site and a nonmagnetic site, and t_2 is hopping integral between d_{xy} orbital at a magnetic site and d_{yz} orbital at a nonmagnetic site. In the inversion symmetric system, t_2 vanishes. Thus t_2 scales the inversion symmetry breaking (ISB) of the trimer model [13].

In calculation of the orbital magnetization $\boldsymbol{M}_\alpha = -\mu_B \langle \boldsymbol{L}_\alpha \rangle$ (μ_B: Bohr magneton, here we use $\hbar = 1$), we take $H_{\text{total}} = H_0(\phi = 0) + H_{\text{SOI}}$ and treat the spin-orbit interaction (SOI) H_{SOI} as a perturbation. From the Bruno's work [5], we have

$$\langle \boldsymbol{L}_\alpha \rangle = \sum_{\text{exc}} \langle \text{gr} | \boldsymbol{L}_\alpha | \text{exc} \rangle \frac{\langle \text{exc} | H_{\text{SOI}}^{\alpha} | \text{gr} \rangle}{E_{\text{gr}} + E_{\text{exc}}} + \text{c.c.} \tag{7}$$

where L_α are the orbital momentum operators, $|\text{gr}\rangle (|\text{exc}\rangle)$ and $E_{\text{gr}}(E_{\text{exc}})$ represent the ground (excited) state and its energy, respectively. Since the spin quantization axis is chosen as the magnetization direction, we have

$$\boldsymbol{M} \| x{:}\ H_{\text{SOI}}^{x} = \frac{\lambda}{2}\left(\boldsymbol{L}_x \boldsymbol{\sigma}_z + \boldsymbol{L}_y \boldsymbol{\sigma}_y - \boldsymbol{L}_z \boldsymbol{\sigma}_x\right), \boldsymbol{M} \| z{:}\ H_{\text{SOI}}^{z} = \frac{\lambda}{2}\left(\boldsymbol{L}_z \boldsymbol{\sigma}_z + \boldsymbol{L}_x \boldsymbol{\sigma}_x + \boldsymbol{L}_y \boldsymbol{\sigma}_y\right) \tag{8}$$

where $\sigma_{x,y,z}$ are the Pauli matrices and λ is the atomic spin-orbit coupling constant.

In the canted magnetization configuration, the SOI gives an energy correction corresponding to the DMI energy. The DMI energy is written as

$$E_{\text{DMI}} = \sum_{\text{exc}} \langle \text{gr} | \Delta V | \text{exc} \rangle \frac{\langle \text{exc} | H_{\text{SOI}} | \text{gr} \rangle}{E_{\text{gr}} + E_{\text{exc}}} + \text{c.c.} \tag{9}$$

where $\Delta V = -Im\phi\ \sigma_x \delta_{AA}$ is a perturbation of a small exchange field corresponding to a canted magnetic configuration (δ_{AA} is a projection operator onto site A). Equation (9) looks similar to the equation for the orbital momentum Eq (7). The main difference between Eq. (7) and Eq. (9) is that ΔV flips spin whereas $\boldsymbol{L}_\alpha$ does not. This similarity implies a possible connection between DMI and orbital moment anisotropy.

In our coordinate notation, *x*- and *y*-axes lie in the plane and *z*-axis along the out of the plane. Henceforth, we denote *x*-direction as ∥ (in-plane) and *z*-direction as ⊥ (perpendicular to the plane).

b. Result

(a) Orbital moment and DMI at zero temperature

For $\phi = 0$, the Hamiltonian H_0 is a block diagonal matrix, $H_0(\phi = 0) = H^{\uparrow\uparrow} \oplus H^{\downarrow\downarrow}$. In majority spin subspace, the eigenvalues E_n $(n = 1, \ldots, 5)$ and corresponding eigenvectors $|n\rangle$ of $H^{\uparrow\uparrow}$ are given as

$$E_1 = \frac{1}{2}(E_A + E_C + J) + \frac{1}{2}W_{+,1}, \quad E_2 = \frac{1}{2}(E_A + E_C + J) - \frac{1}{2}W_{+,1},$$
$$E_3 = \frac{1}{2}(E_A + E_C + J) + \frac{1}{2}W_{+,2}, \quad E_4 = \frac{1}{2}(E_A + E_C + J) - \frac{1}{2}W_{+,2}, \quad E_5 = E_C, \tag{10}$$

$$|1\rangle = \sqrt{\frac{1}{2 + \frac{4t_1^2}{E_1 - E_C}}}(1, 1, \frac{2t_1}{E_1 - E_C}, 0, 0)^T, \qquad |2\rangle = \sqrt{\frac{1}{2 + \frac{4t_1^2}{E_2 - E_C}}}(1, 1, \frac{2t_1}{E_2 - E_C}, 0, 0)^T,$$
$$|3\rangle = \sqrt{\frac{1}{2 + \frac{4t_2^2}{E_3 - E_C}}}(1, -1, \frac{2t_2}{E_3 - E_C}, 0, 0)^T, \qquad |4\rangle = \sqrt{\frac{1}{2 + \frac{4t_2^2}{E_4 - E_C}}}(1, -1, \frac{2t_2}{E_4 - E_C}, 0, 0)^T,$$
$$|5\rangle = (0, 0, 0, 0, 1)^T \tag{11}$$

where

$$W_{\pm,1} = \sqrt{E_\pm^2 + 8t_1^2}, \quad W_{\pm,2} = \sqrt{E_\pm^2 + 8t_2^2}, \quad E_\pm = E_A - E_C \pm J, \quad J = \frac{1}{2}Im. \tag{12}$$

Replacing J of Eqs. (10) and (11) with $-J$, we obtain eigenvalues and corresponding eigenvectors of $H^{\downarrow\downarrow}$. Using E_n and $|n\rangle$, we obtain the orbital magnetizations and DMI energies in terms of the

occupation number N_e. From Eqs. (7), (10) and (11), we easily read that $\langle \boldsymbol{L}_\alpha \rangle$ results from hopping between d_{xy} orbital at magnetic site and the same orbital at nonmagnetic site ($\langle 1|\boldsymbol{L}_x|5\rangle, \langle 2|\boldsymbol{L}_x|5\rangle$), which is not related to the ISB parameter. On the other hand, $\langle \boldsymbol{L}_z \rangle$ results from hopping between d_{xy} at a magnetic site and d_{yz} at a nonmagnetic site ($\langle 3|\boldsymbol{L}_z|5\rangle, \langle 4|\boldsymbol{L}_z|5\rangle$), which originates from the ISB parameter. Therefore, $\langle \boldsymbol{L}_\parallel \rangle (\langle \boldsymbol{L}_\perp \rangle)$ depends only on $t_1 (t_2)$ in our model. We choose $N_e = 8$ in this work. This electronic configuration corresponds to fully occupied majority band and two unoccupied states in the minority band, which closely mimics the PDOS of Co/Pt. By computing possible quantum transitions for $N_e = 8$ and assuming $t_1 > t_2$, we obtain

$$\langle \boldsymbol{L}_\parallel \rangle = \lambda\left(\frac{1}{W_{+,1}} + \frac{E_+ - W_{+,1}}{4t_1^2}\right), \langle \boldsymbol{L}_\perp \rangle = \lambda\left(\frac{1}{W_{+,2}} + \frac{E_+ - W_{+,2}}{4t_2^2}\right)$$

$$E_{\mathrm{DMI}} = -J\phi\lambda t_1 t_2 \left\{\frac{2(t_1^2 - t_2^2) + JE_+ - JW_{+,1}}{2W_{+,1}[(t_1^2 - t_2^2)^2 + J(t_1^2 E_- - t_2^2 E_+)]} + \frac{2(t_1^2 - t_2^2) + JE_+ - JW_{+,2}}{2W_{+,2}[(t_1^2 - t_2^2)^2 + J(t_1^2 E_+ - t_2^2 E_-)]}\right\}. \quad (13)$$

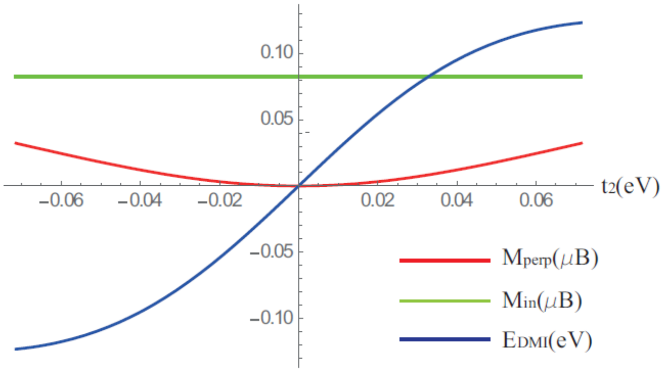

Supplementary Figure 8. Tight binding model calculation results. Orbital magnetizations ($M_\alpha = -\mu_B \langle L_\alpha \rangle$) and DMI as functions of ISB parameter t_2. The parameters $t_1 = 0.5$ eV, $E_A = 0$ eV, $E_C = 0.5$ eV, $I = 0.96$ eV, $m = 1.8\mu_B$, and $\lambda = 0.2$ have used. For the DMI energy calculation, we chose $\phi = \pi/4$.

Supplementary Figure 8 shows that $\boldsymbol{L}_\perp(E_{\mathrm{DMI}})$ is semi-quadratic (linear) in t_2 whereas $\langle \boldsymbol{L}_\parallel \rangle$ does not depend on t_2. Therefore the ISB parameter t_2 induces $\langle \boldsymbol{L}_\perp \rangle$ and E_{DMI}. For $t_2 \ll 1$ and $E_+ > 0$, the equations in (13) are further simplified;

$$\langle \boldsymbol{L}_\parallel \rangle = \lambda\left(\frac{1}{W_{+,1}} + \frac{E_+ - W_{+,1}}{4t_1^2}\right), \quad \langle \boldsymbol{L}_\perp \rangle = \lambda\left(\frac{2t_2^2}{E_+^3}\right),$$

$$E_{\mathrm{DMI}} \approx -J\phi\lambda t_1 t_2 \left\{\frac{2t_1^2 + J(E_+ - JW_{+,1})}{2t_1^2(JE_- + t_1^2)W_{+,1}} - \frac{1}{(JE_+ - t_1^2)E_+}\right\}. \quad (14)$$

We note that t_2 -dependence is explicitly described in Eq. (14) (i.e., $W_{\pm,1}$ - and $E_{\pm}$ -related terms do not include t_2). The linear dependence of DMI on t_2 is consistent with Ref. [14] which describes the DMI from the viewpoint of Rashba Hamiltonian with sd exchange. From the perturbation description of the Rashba theories [14-17], we expect that the Rashba constant of our model is $\alpha_R \propto \lambda t_2 / \Delta$ ($\Delta = E_C - E_A$) [18]. On the other hand, the perpendicular orbital magnetization shows semi-quadratic dependence on t_2 .

c. Temperature dependence of orbital moment, spin density distribution, and DMI energy

In this subsection, based on the trimer model, we show a numerical result of the temperature dependence of orbital moment and DMI energy with respect to full order calculation in spin-orbit coupling parameter λ. In order to describe the temperature dependence in our trimer model, we assume that the increase in temperature leads to the increase in broadening of density of state (DOS). As discussed in the main manuscript, this broadening reflects the magnetization fluctuations and atomic vibrations (electron-phonon interaction) effectively. We note that we restrict our aim at a qualitative description to the orbital moment and DMI energy in this simple model description. The equations for orbital moment and the DMI energy are given as

$$\langle \boldsymbol{L} \rangle = \sum_m \int dE\, f_{\mathrm{FD}}(E) \boldsymbol{L}_{\mathrm{m}} n_{\mathrm{m}}(E), \quad E_{\mathrm{DMI}} = \sum_m \int dE\, f_{\mathrm{FD}}(E) H_{\mathrm{SOI,m}} n_{\mathrm{m}}(E), \tag{15}$$

where $f_{FD}(E)$ is the Fermi-Dirac distribution function, L_m and $H_{SOI,m}$ are expectation values of the angular momentum and the spin-orbit interaction matrices in the m-th eigenstate, respectively. $n_{\mathrm{m}}(E) = (1/\pi)\cdot(\Gamma / [(E - E_{\mathrm{m}})^2 - \Gamma^2])$ is the density of state corresponding to m-th energy eigenvalue E_{m}. The temperature effect is considered in the Fermi-Dirac distribution function and the Lorentzian broadening Γ. For temperature dependence of Γ, we assume that Γ(T)=0.5T-35 meV for (100K≤T≤300K). Here, we introduce a proximity-induced small magnetization on the nonmagnetic site. Note that this additi onal magnetization does not alter the conclusion deduced from Supplementary Figure 8, but changes the n umerical values of orbital moments and DMI energy. The ratio between magnetic moment on nonmagneti c site and magnetic site is set to 1/10. For $N_e = 8$, we depict the temperature dependence of the orbital m oments and DMI in Fig. 5b of the main manuscript. The parameters are $t_1 = 0.5$ eV, $t_2 = -0.35$ eV, $E_A = 0$ eV, $E_C = 1$ eV, $m = 1.8\mu_B$, $I = 0.96$ eV, and $\lambda = 0.6$. For the DMI energy calculation, $\phi = \pi/4$ has chosen.

Supplementary Note 7.

Detail discussions about the correlation of DMI with the orbital anisotropy and m_D based on the *ab-initio* calculation

In order to demonstrate the observed correlation between the DMI, orbital moment anisotropy (OMA), and magnetic dipole moment operator $<T_z>$ ($=m_D/7$ in the main text) and elucidate their physical origin we have performed density-functional theory calculations using the full-potential linearized augmented plane wave method as implemented in the FLEUR code [19]. Based on the experimental observation, the microscopic origin of this correlation clearly involves the impact of the temperature on 3*d*-orbital magnetization and their electron filling, which affects significantly the magnetic coupling between the 3d orbitals of the Co overlayer and the 5*d* orbitals of the Pt substrate. In this respect, to explain this effect from an ab-initio point of view, it is instructive to vary the 3*d* transition metals (TMs) on the Pt substrate and examine the general chemical trend. Indeed, it is helpful to deal with different 3*d* TMs on Pt(111) surface in order to understand how the 3*d* orbital occupations can affect various magnetic quantities related to inversion symmetry breaking at interface.

In Figure S6a-S6b, the total DMI energy D^{tot} and OMA in 3*d*/Pt(111) are represented as a function of the 3*d* overlayers (V, Cr, Mn, Fe, Co, Ni). Notice that the OMA reported on Supplementary Figure 9b is the total OMA that includes both contribution the 3*d* overlayer and Pt substrate. We find interesting trends across the 3*d* series considered in our study for which the strength and sign of DMI clearly correlated with the OMA. As we can see in Supplementary Figure 9a and 9b the majority of 3*d*/Pt(111) interfaces have a positive sign of D^{tot} and OMA, while for V/Pt(111) the OMA is negative which reverses the sign of DMI from left- to right-chirality. The largest absolute values are obtained for Mn overlayer [~ 9 meV nm/Mn atom and 1.35 for D^{tot} and OMA, respectively] and they gradually decrease towards V or Ni atoms. Indeed, the trends found in 3*d*/Pt(111) interfaces are consistent with the behavior observed in low-dimensional systems whose spin moments as function of the number of *d* electrons are well described by Hund's first rule see Supplementary Figure 9d (orange line). On the other hand, the degree of hybridization between the ferromagnet and substrate states is tuned upon varying the 3*d* orbitals filling, such that weak hybridization enhances the importance of the SOC and vice versa [20-22], e.g., for Mn overlayer both of spin channels up (down) are occupied (unoccupied) and therefore a weak hybridization with the substrate states will enhance not only the SOC effects in Mn/Pt system but also the degree of symmetry breaking at the interface in comparison with other investigated systems. The latter was confirmed previously for the trend of the electric dipole moment and degree of hybridization across 3*d*/5*d* interfaces [21]. In this context, the orbital anisotropy ratio should be larger for Mn and decreases gradually for 3*d* elements on both sides as discussed above [See Supplementary Figure 9b]. Obviously,

the DMI shows no direct correlation with the proximity induced magnetism in Pt across the 3*d*/Pt interfaces [Supplementary Figure 9d (gray line)], since the 5*d* states are degenerate and partially occupied due the strong crystal-field splitting, irrespective of deposited 3*d* overlayer on the top. This is in good agreement with the experimental observations. However, the presence of the 5*d* states of the Pt substrate around the Fermi energy facilitates the spin-flip excitation mechanism needed for the DMI as well as influences the intra-atomic magnetic dipole moment $<\boldsymbol{T}_z>$. Physically, the $<\boldsymbol{T}_z>$ term reflects the anisotropy or asphericity the spin density distribution (SDD) due to the breaking of crystal symmetry [23-25]. As shown in Supplementary Table 2, $<\boldsymbol{T}_z>$ depends on the magnetic quantum number *lm* and the matrix elements of quadrupole moment operator $\langle Y_{2m}|\hat{Q}|Y_{2m}\rangle$. The latter can be defined on the basis of real spherical harmonics in DFT. One needs to integrate the electron densities projected on those spherical harmonics to obtain the magnetic moment for each component of the d-orbitals. Accordingly, the $\boldsymbol{T}_z$ term depends on the competition between the in-plane (d_{xy}, d_{x2-y2}) and out-of-plane (d_{z2}, d_{xz}, d_{yz}) SDD of d-orbital components (see Supplementary Table 2). Hence, a strong $<\boldsymbol{T}_z>$ reflects the large difference between the occupation of in-plane and out-of-plane orbitals. Note that if SDD is spherically symmetric like in the bulk systems then $<\boldsymbol{T}_z>$ should vanish. It is also worthwhile noticing that $<\boldsymbol{T}_z>$ is not only the consequence of inversion symmetry breaking and reduction in atomic coordination number, but also depends on the atomic nature of the ferromagnet (it can appear even in the absence of SOC). In other words, the intra-atomic dipole operator establishes a close link between the orbital magnetism and spin excitation in term of electron hopping, and a detailed understanding of this quantity could explain the physical mechanism behind the strong correlation. More specifically, by varying the occupation of the 3*d* overlayer shell the magnetic dipole moment $<\boldsymbol{T}_z>$ also changes, which is crucial for the appearance of both orbital anisotropy and DMI. They can be easily influenced by the strength of the interfacial 3*d*-5*d* hybridization [21-23]. This can be understood by examining the energetic positions of 3*d*/5*d* orbitals and their SDD upon hybridization. This fact is reflected, for instance, by the trend of $<\boldsymbol{T}_z>$ and effective spin moment as a function of the 3d orbital filling, as displayed in Supplementary Figure 9, see also Supplementary Table 2. As expected, we find a pronounced correlation between the magnetic dipole moment and DMI, indicating that the physical mechanism responsible for DMI is intimately related to filling of 3*d* TMs and their orbital magnetization explained in term of the anisotropy of SDD between the components of 3d orbital. According to Supplementary Figure 9, the largest absolute $7<\boldsymbol{T}_z>$ (=$\boldsymbol{m}_D$ in the main text) value is obtained for Mn/Pt interface 0.75 μ_B/atom, which is larger compared to only 0.17 and 0.03 μ_B/atom for Ni/Pt and V/Pt, respectively. Furthermore, this correlation happens not only for the total $<\boldsymbol{T}_z>$ but also for both the orbital magnetic moment for each *d*-component and the resulting effective spin moment ($\boldsymbol{m}_{eff}$). Their variations follow perfectly Hund's first rule. (See Supplementary Figure 9c and 9d (yellow line) and Supplementary Table 2).

More interestingly, the chemical trend of <$\boldsymbol{T}_z$> shows a clear dependence on both states occupied and unoccupied spin channels of d-orbitals, e.g., the case of Mn/Pt, which reflect the large magnetic moment and stronger orbital anisotropy, resulting in a large DMI. This holds true not only for <$\boldsymbol{T}_z$> but also for DMI since both of states should be available to facilitate the electron hopping between the d orbitals, and consequently allow the spin-flip excitation processes needed for DMI. An analogue behavior has been found for Co/5d and Co/Pd by Sipr *et al*. [21,22] and Wu *et al*. [23], who emphasized the importance of the unoccupied minority spin channel, which induces a large asphericity of SDD and a strong <$\boldsymbol{T}_z$>.

Supplementary Table 2. Calculated magnetic dipole moment $\boldsymbol{m}_D$=7<$\boldsymbol{T}_z$> (using the approximate relation $\boldsymbol{T}_\alpha = \sum_m 1/2\langle Y_{2m}|\hat{Q}|Y_{2m}\rangle\mu_{spin}^m$) of 3d transition-metal monolayers on Pt (111) surface.

	<$\boldsymbol{T}_z$> [3d/Pt(111)]					
Component	Ni	Co	Fe	Mn	Cr	V
xy	0.1255	0.2453	0.3606	0.3966	0.2559	0.043
yz	-0.0682	-0.1191	-0.1846	-0.2145	-0.1371	-0.0227
zx	-0.0685	-0.119	-0.1846	-0.2139	-0.1371	-0.0228
x^2-y^2	0.1261	0.2453	0.3611	0.395	0.2564	0.043
z^2	-0.1404	-0.3113	-0.4487	-0.4711	-0.3272	-0.04593
Sum	-0.0255	-0.0588	-0.0962	-0.108	-0.0892	-0.0048
7<$\boldsymbol{T}_z$>	-0.1785	-0.412	-0.6735	-0.7562	-0.6245	-0.0339
$\boldsymbol{m}_{\mathrm{eff}}$	0.7084	1.6199	2.3264	3.0337	2.3754	1.6199

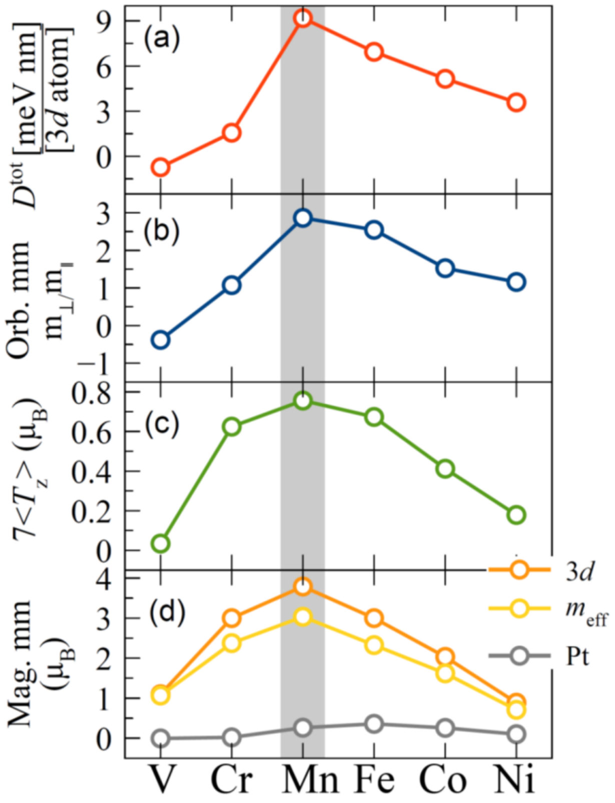

Supplementary Figure 9. The *ab-initio* calculation results. a. Strength and sign of $\boldsymbol{D}^{tot}$ in 3*d* transition-metal monolayers on Pt (111) substrate calculated around their magnetic ground state using the combination of the relativistic effect spin-orbit coupling with the spin spirals. A positive sign of $\boldsymbol{D}^{tot}$ indicates a left-rotational sense or "left chirality". **b.** $\boldsymbol{m}_o^{\perp}/\boldsymbol{m}_o^{\parallel}$, **c.** 7<$T_z$>, and **d.** the effective magnetic moment ($\boldsymbol{m}_{eff}$) and the magnetic moments per 3*d* and Pt atoms.

Reference

[1] Moriz, J., *et al.* Experimental evidence of a activation law in nanostructures with perpendicular magnetic anisotropy. *Phys. Rev. B* **71**, 100402(R) (2005).

[2] Je, S.-G., *et al.* Asymmetric magnetic domain-wall motion by the Dzyaloshinskii-Moriya interaction. *Phys Rev B* **88**, 214401 (2013).

[3] Pizzini, S., *et al.* Chirality-Induced Asymmetric Magnetic Nucleation in Ultrathin Microstructures. *Phys. Rev. Lett.* **113**, 047203 (2014).

[4] Kim, S. *et al.* Magnetic droplet nucleation with a homochiral Néel domain wall. *Phys Rev B* **95**, 220402(R) (2017).

[5] E. Weinan, W. Ren, and E. Vanden-Eijnden, Simplified and improved string method for computing the minimum energy paths in barrier-crossing events. *J. Chem. Phys.* **126**, 164103 (2007).

[6] Chaves-O'Flynn, G. D., Bedau, D., Vanden-Eijnden, E., Kent, A. D., & Stein, D. L Stability of 2π Domain Walls in Ferromagnetic Nanorings. *IEEE Trans. Magn.* **46**, 2272-2274 (2010).

[7] Nembach, H. T., Shaw, J. M., Weiler, M., Jué, E., & Silva, T. J. Linear relation between Heisenberg exchange and interfacial Dzyaloshinskii–Moriya interaction in metal films. *Nat. Phys.* 11, 825-829 (2015).

[8] Bruno, P. Tight-binding approach to the orbital magnetic moment and magnetocrystalline anisotropy of transition-metal monolayers. *Phys., Rev. B* **39**, 865-868 (1989).

[9] Thole, B. T., Carra, P., Sette, F. & van der Laan, G. X-ray circular dichroism as a probe of orbital magnetization. *Phys. Rev. Lett.* **68**, 1943-1946 (1992).

[10] Carra, P., Thole, B. T., Altarelli, M. & Wang, X. X-ray circular dichroism and local magnetic fields, *Phys. Rev. Lett.* **70**, 694-697 (1993).

[11] Wu, R. & Freeman, A. J. Limitation of the magnetic-circular-dichroism spin sum rule for transition metals and importance of the magnetic dipole term. *Phys. Rev. Lett.* **73**, 1994-1997 (1994).

[12] König, H. & Stöhr, J. Determination of spin-and orbital-moment anisotropies in transition metals by angle-dependent X-ray magnetic circular dichroism. *Phys. Rev. Lett.* **75**, 3748-3751 (1995).

[13] Kashid, V. *et al.* Dzyaloshinskii-Moriya interaction and chiral magnetism in 3*d*-5*d* zigzag chains: Tight-binding model and *ab initio* calculations. *Phys. Rev. B* **90**, 054412 (2014).

[14] Kim, K. -W. *et al*. Chirality from interfacial spin-orbit coupling effects in magnetic bilayers. *Phys. Rev. Lett*. **111**, 216601 (2013).

[15] Petersen, L. *et al*. Petersen, Lars, and Per Hedegård. A simple tight-binding model of spin–orbit splitting of sp-derived surface states. *Surf. Sci.* **459**, 49-56 (2000).

[16 Kim, Y., Lutchyn, R. M., & Nayak, C. Origin and transport signatures of spin-orbit interactions in

one-and two-dimensional SrTiO 3-based heterostructures. *Phys. Rev. B* **87**, 245121 (2013).

[17] Kim, P., Kang, K. T., Go, G., & Han, J. H. Nature of orbital and spin Rashba coupling in the surface bands of $SrTiO_3$ and $KTaO_3$. *Phys. Rev. B* **90**, 205423 (2014).

[18] Since our model is based on atomic trimer which has no Bloch momentum, the Rashba constant of the trimer model is not valid. However, if we extend our model to two-dimensional plane with an ISB parameter t_2, the resultant Rashba constant is proportional to $\lambda t_2 / \Delta$.

[19] URL http://www.flapw.de.

[20] A. Belabbes *et al*. Hund's Rule-Driven Dzyaloshinskii-Moriya Interaction at Interfaces. *Phys. Rev. Lett.* **117**, 247202 (2016).

[21] O. Sipr *et al*. Co monolayers and adatoms on Pd (100), Pd (111), and Pd (110): Anisotropy of magnetic properties. *Phys. Rev. B* **88**, 064411 (2013).

[22] Šipr, O., Minár, J., & Ebert, H. Influence of spin-orbit coupling on the magnetic dipole term Tα. *Phys. Rev. B* **94**, 144406 (2016).

[23] Wu, R. and Freeman, A. J. Limitation of the magnetic-circular-dichroism spin sum rule for transition metals and importance of the magnetic dipole term. *Phys. Rev. Lett.* **73**, 1994 (1994).

[24] Šipr, O., Minár, J., & Ebert, H. On the importance of the magnetic dipole term Tz in analyzing X-ray magnetic circular dichroism spectra of clusters. *Europhys. Lett.* **87**, 67007 (2009).

[25] Oguchi, T. & Shishidou, T. Anisotropic property of magnetic dipole in bulk, surface, and overlayer systems. *Phys. Rev. B* **70**, 024412 (2004).
